# Stochastic resilience of Arctic sea ice: a framework bridging theory and observation

**DOI:** 10.1038/s41612-026-01455-z

**Published:** 2026-06-15

**Authors:** Chuixiang Yi, Lu Zhou, Deliang Chen

**Affiliations:** 1 School of Earth and Environmental Sciences, Queens College, City University of New York, New York, NY USA; 2 Earth and Environmental Sciences Department, Graduate Center, City University of New York, New York, NY USA; 3 Institute for Marine and Atmospheric Research, Utrecht University, Utrecht, Netherlands; 4 Department of Earth System Science, Tsinghua University, Beijing, China; 5 Institute for Carbon Neutrality, Tsinghua University, Beijing, China

**Keywords:** Climate sciences, Ocean sciences

## Abstract

Arctic sea ice has declined rapidly in extent, thickness, and albedo, yet a quantitative observational measure of its resilience remains elusive. Here, we introduce a resilience potential framework that links a reduced Arctic surface energy balance to an effective potential whose curvature and barrier structure provide physically interpretable stability diagnostics. Using four decades of sea ice extent, thickness, and albedo, we compare contextual diagnostics of recovery and variability with empirical potential analogs inferred from October sea ice volume-derived energy states. The empirical reconstruction reveals a shift of the occupied ice-covered state toward reduced latent energy storage, while bootstrap tests show that the recent-period barrier is not robustly identifiable and is more consistently represented as a single empirical well under the adopted preprocessing assumptions. Our results, therefore, show how a physically based potential framework can be used to interpret aggregate sea ice variability and to test alternative hypotheses about changing resilience. This framework provides a general, observation-based pathway for quantifying resilience in Arctic sea ice and, more broadly, other Earth system components.

## Introduction

The Arctic is warming nearly four times faster than the global mean^1^, driving an accelerating decline in sea ice cover^2,3^. As a key regulator of the Earth’s energy balance^4,5^, sea ice reflects incoming solar radiation while insulating the ocean from the atmosphere^6–9^. Through this dual role, Arctic sea ice shapes polar climate and exerts far-reaching influence on weather patterns and ecosystems well beyond the high latitudes. Over the past four decades, satellite observations have documented a sustained and accelerating loss of Arctic sea ice extent (SIE) and thickness (SIT)^10–13^. This long-term decline has been punctuated by abrupt changes, most notably around 2007, when the ice cover shifted from a thicker, mechanically deformed, multi-year regime to a thinner and more homogeneous state^14,15^.

A substantial body of work argues that summer Arctic sea ice loss is more consistent with a largely quasi-linear forced response than with a deterministic bistable tipping point^16,17^. In particular, stabilizing feedbacks associated with longwave cooling, the seasonal cycle, and the absence of a stable “summer ice” equilibrium in many models can weaken or eliminate classical critical slowing down (CSD) signatures; moreover, in some frameworks CSD is not expected before bifurcation^16–18^, so the interpretation of early-warning indicators from observations remains nuanced. The prevailing assessment, also reflected in the Global Tipping Points Report 2025^19^, is that deterministic bistability in summer sea ice is very unlikely, while winter stability remains more uncertain because volume integrates multi-seasonal memory and dynamical redistribution. In this context, any potential-well interpretation inferred from observations must be tested against alternative explanations, including a single drifting stable basin under forced decline with state-dependent variance and memory, and statistical artefacts arising from bounded or trending data^20,21^.

At the same time, the ice-albedo feedback remains a physically important nonlinearity: as ice melts, darker ocean surfaces absorb more solar radiation, enhancing ocean warming and accelerating further melt^9^. In reduced models and under some climate states, this feedback may permit multiple equilibria or abrupt transitions between contrasting regimes^22–26^. Paleoclimate records and numerical experiments further suggest that threshold-like Arctic sea ice changes have occurred in past climates^27–31^. These results motivate testing reduced nonlinear frameworks against observations, while keeping in view that such behavior is not the current consensus interpretation for the modern summer Arctic.

Despite these insights, the proximity of the present-day Arctic sea ice system to a tipping point and the degree to which it retains resilience, defined as its capacity to absorb perturbations without shifting to a new regime, remain deeply uncertain^32–37^. While simplified energy balance models have provided valuable conceptual understanding, many rely on idealized representations of albedo changes and neglect gradual processes such as melt pond formation, snow metamorphism, and partial ice cover^16,38–40^. Moreover, the role of stochastic variability associated with weather and ocean dynamics is often underrepresented^40^. Such variability can trigger large excursions even before deterministic thresholds are reached, a phenomenon known as noise-induced tipping^41–44^. Prior observational studies have applied critical slowing down and related methods to Arctic sea ice over roughly three decades and did not find clear evidence for bistability in the available record; whether the extended record and alternative stability metrics support or qualify those interpretations requires reassessment^20,21^.

To address these gaps, we introduce a quantitative framework that links the physical energy balance of Arctic sea ice to its stability and tipping behavior. Building on stochastic energy-balance theory, we represent sea ice changes using a continuous, energy-dependent albedo that captures gradual melt processes and regime transitions. This formulation yields a resilience potential landscape, offering intuitive model-based diagnostics of recovery rate, barrier structure, and long-term persistence. By explicitly incorporating stochastic forcing from atmospheric and oceanic variability, we quantify how internal fluctuations erode resilience and increase tipping likelihood. We then confront these theoretical resilience diagnostics with sea ice extent and thickness observations, using the observational record to test which potential-based signatures are supported and to constrain alternative explanations for changing Arctic sea ice resilience. The framework is deliberately reduced to a basin-mean energy balance and cannot represent regional heterogeneity, ice dynamics, or spatially localized feedbacks; its value lies in aggregate stability diagnostics and their linkage to observation-derived potential analogs.

In this study, we contribute three new elements. (1) We present a resilience-potential inference pipeline that links an energy-balance potential (curvature and barrier) to observation-derived analogs inferred from satellite-based sea ice records. (2) We apply this framework to four decades synthesis (1979–2024) of sea ice extent, volume and albedo, enabling reassessment of post-2012 behavior in a consistent processing chain. (3) We perform robustness and alternative-hypothesis tests, including epoch choice, detrending choices, and synthetic monostable controls, to quantify when an apparent barrier is robust versus when it can be produced artefactually.

## Results

### Observed decline in Arctic sea ice albedo, extent and volume

Satellite passive microwave data, providing a consistent pan-Arctic record since 1979, show that September SIE, the annual minimum and a key indicator of system state, has decreased by roughly 40%^3,45^, corresponding to an average loss of 82,000 km^2^ per year. This long-term decline has been punctuated by dramatic record lows, most notably in 2007, 2012, and 2020^45–47^. The anomaly time series suggests a transition to persistently below-average late-summer ice conditions after the mid-1990s^45,48–50^

**Fig. 1:**
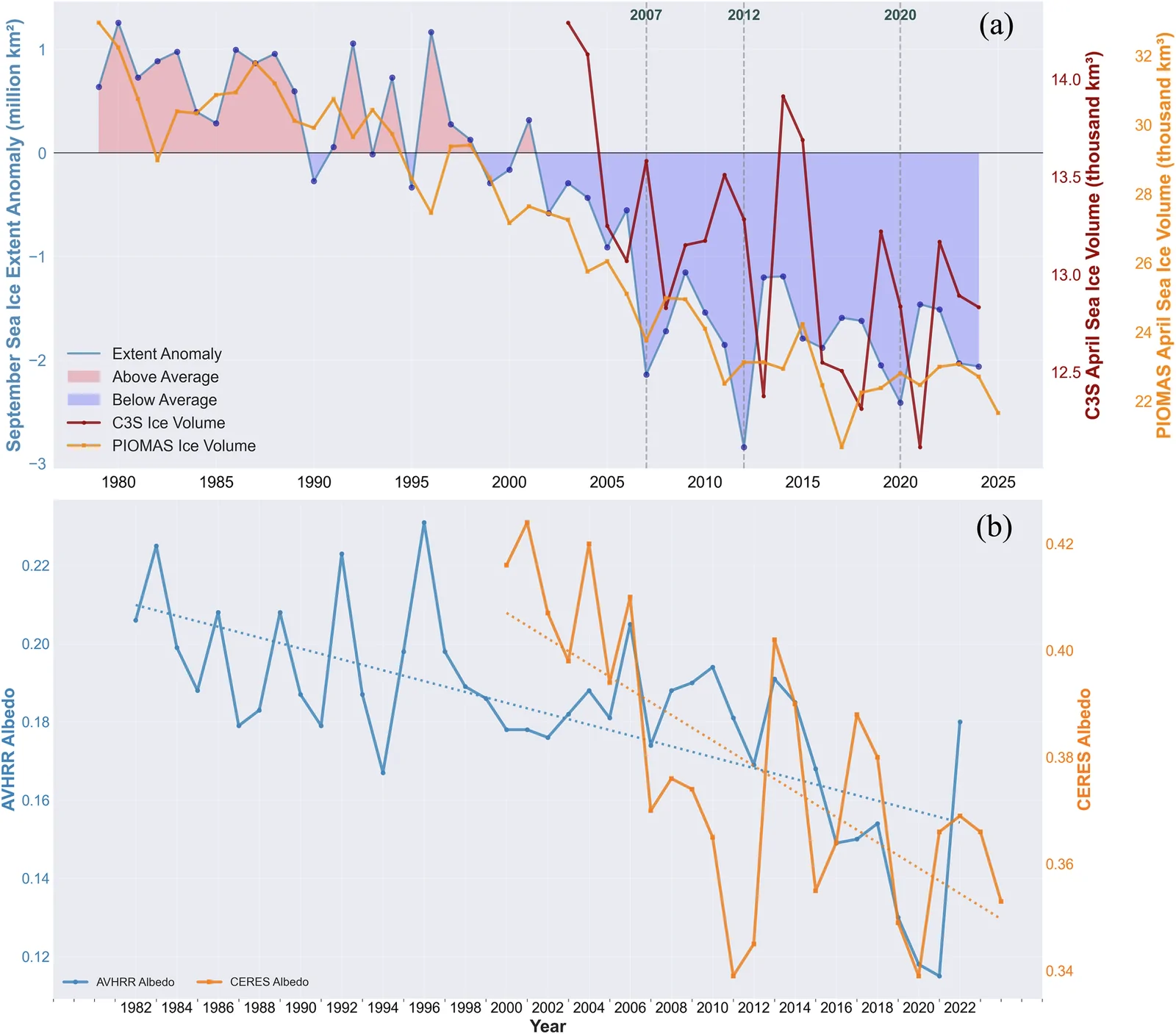
Satellite observations reveal a multi-decadal deterioration of the Arctic sea ice system, reflected consistently across extent, volume, and surface albedo. **a** September sea ice extent anomaly (blue) has decreased by ~ 40% since 1979, with a transition to persistently below-average late-summer conditions after the mid-1990s. Superimposed reconstructions of late-summer sea ice volume (red, C3S; orange, PIOMAS) show a parallel thinning, with total volume dropping from above 13,000 km^3^ in the 1980s to below 12,000 km^3^ in the 2020s. Together these metrics highlight both a long-term forced decline and episodic abrupt losses. **b** Broadband surface albedo during the melt season (July–August) shows a substantial loss of reflectivity. CERES data (orange) indicate a ~ 15% decline since 2000, consistent with the longer AVHRR record (blue). This decline reflects the ice-albedo feedback, whereby thinning ice and prolonged open water seasons amplify solar absorption, compounding volume and extent losses.

Concurrently, the Arctic Ocean’s broadband albedo has declined as open water fractions expanded and multi-year ice retreated. CERES observations^51^ indicate that mean July-August sea ice surface albedo decreased from 0.42 in 2000 to 0.35 in 2024, representing a 15% reduction in regional reflectivity (Fig. 1b). The largest decreases occur in the Beaufort, Chukchi, and Laptev seas, where earlier melt onset, loss of multi-year ice, and prolonged open water seasons have lowered local albedo by more than 0.20^3,52^. These radiative losses reinforce the icealbedo feedback, whereby reduced reflectivity enhances ocean heat uptake and accelerates further melt^8,22^.

Losses in extent and albedo are compounded by substantial thinning of the sea ice cover^23^. Reconstructions of sea ice volume using the coupled ice-ocean model PIOMAS^53,54^ indicate that September ice volume has declined by 10% since the early 2000s (Fig. 1a), consistent with estimates from satellite altimetry [e.g., ICESat and CryoSat-2^12,55^]. Mean September SIV has decreased above 13,000 km^3^ in the 1980s to roughly 12,000 km^3^ in the 2020s^56,57^.

These changes are not purely gradual but intermittently amplified by extreme events that expose the system’s fragility^58^. The 2012 record minimum, for example, was driven in part by an intense August cyclone that fragmented an already weakened ice cover following an early melt onset^59–61^, while the 2020 minimum followed an extreme heatwave across north Siberia^44^ and anomalously strong melt in the Kara and Laptev Seas^62^. Such episodic perturbations interact with long-term forcing to shape variability and recovery, underscoring the need for a framework that captures both gradual decline and abrupt excursions in Arctic sea ice stability^17,63–65^.

### A resilience potential framework links energy balance to observation-derived stability proxies

The observed long-term decline and episodic extremes of Arctic sea ice indicate that its evolution is not always well summarized by a single linear trend, thus requiring the further tests of nonlinear stability interpretations. Instead, the system exhibits memory, feedback, and abrupt excursions characteristic of nonlinear dynamics. To capture this behavior, we use a unified energy balance framework that represents both ice-covered and ice-free conditions through a continuous state variable, *E*, describing the net surface energy of the Arctic sea ice (Methods). When the system is ice-covered (*E* < 0), this variable represents latent heat stored in the ice pack and scales directly with sea ice thickness (*hi*). When the system is ice-free (*E* > 0), it represents sensible heat stored in the upper ocean mixed layer, influencing upper ocean temperature.

Integrating the surface energy balance yields a resilience potential *U*(*E*) that quantifies the stability of each regime under external forcing (Methods). In this idealized energy balance model (EBM), the resulting potential contains two stable minima corresponding to a perennial ice-covered state and a summer ice-free state, separated by an unstable threshold near *E* = 0 (Supplementary Fig. 3). Within the model, the barrier height (Δ*U*) provides a model-defined diagnostic of resistance to transitions between states, while the curvature (${{\rm{U}}}^{{\prime\prime} }$ (*E*)) around each minimum reflects the rate of local recovery toward equilibrium, with steeper curvature indicating faster recovery.

The key nonlinearity arises from the albedo energy feedback, represented by a smooth and continuous sigmoid function *α*(*E*) (Supplementary Fig. 1). Compared with earlier stepwise parameterizations of surface albedo^16^, this formulation avoids artificial discontinuities and reflects the continuous transition between ice-covered and ice-free conditions, enabling the model to represent observed coupling between melt dynamics and radiative feedback. This bistable structure captures the essence of Arctic vulnerability: the perennial ice-covered regime remains present, but it is no longer uniquely stable. Large stochastic anomalies, such as a warm summer or an intense cyclone, can push the system across the unstable ridge, producing an abrupt shift even without sustained forcing changes.

**Fig. 2:**
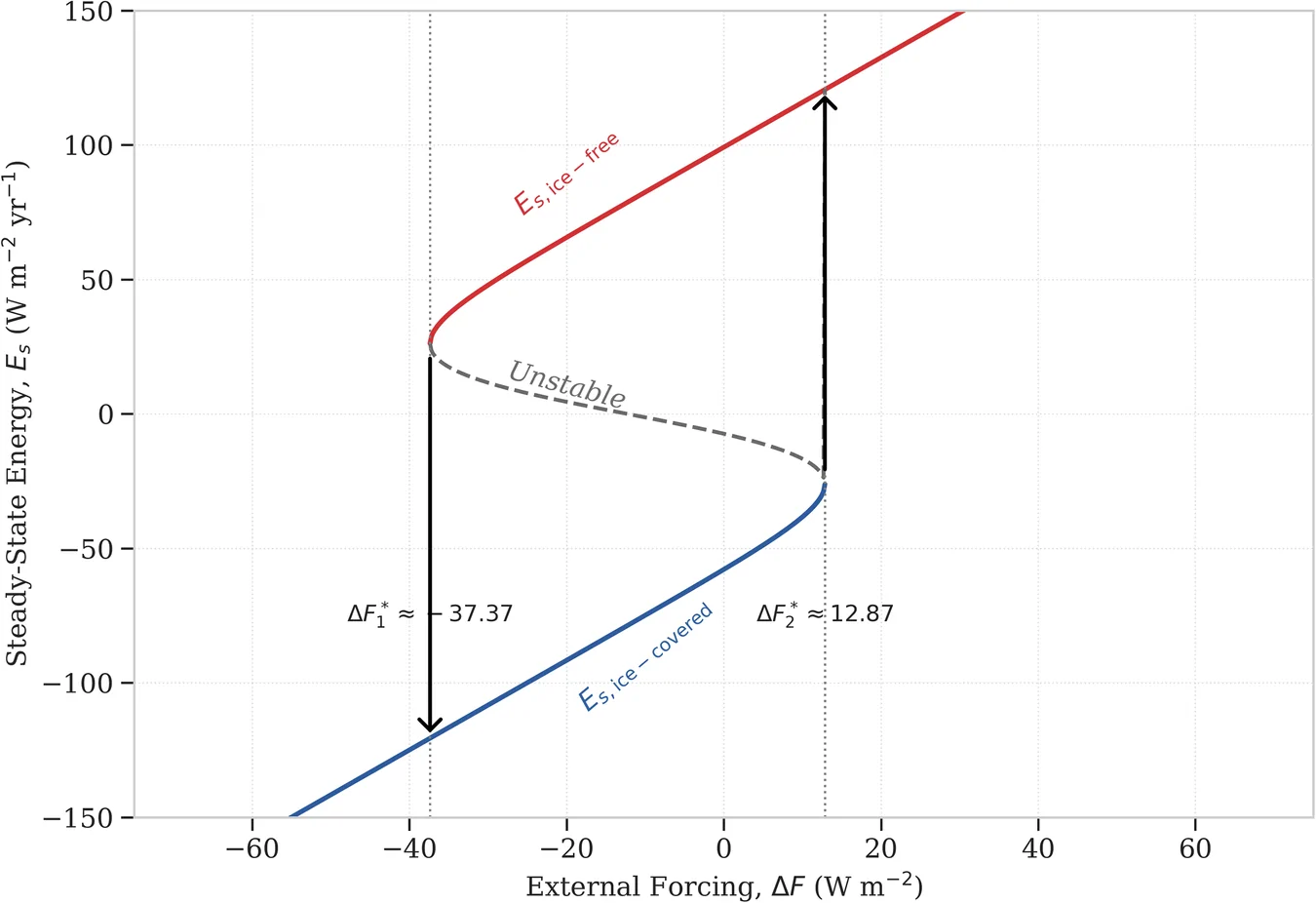
Hysteresis and tipping in the Arctic sea ice model. The bifurcation diagram shows stable ice-covered and ice-free states (solid lines) and the unstable threshold (dashed line) as a function of forcing, Δ*F*, which crosses the bifurcation thresholds $\Delta {F}_{1}^{* }$ and $\Delta {F}_{2}^{* }$.

**Fig. 3:**
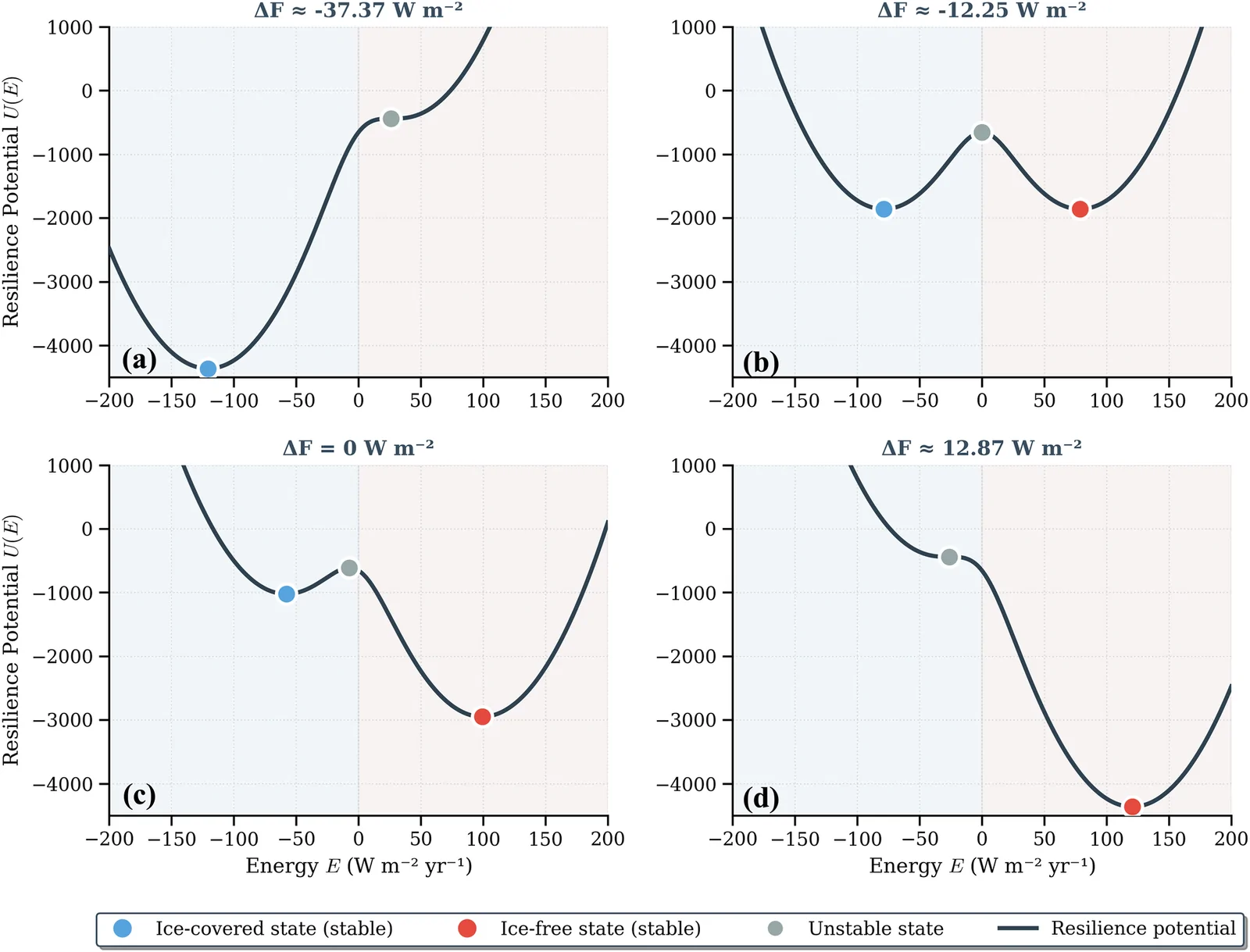
Evolution of the resilience potential under rising radiative forcing. The panels show how the resilience potential of the Arctic sea ice system changes as external forcing (ΔF) increases. **a** At the first tipping point (Δ*F* ≈ − 37.37 W m^−2^), the system is monostable with a single, deep ice-covered valley. **b** At intermediate negative forcing (Δ*F* = − 12.25 W m^−2^), the system is bistable, with both ice-covered and ice-free local minima separated by an unstable ridge. **c** With no external forcing (*ΔF* = 0 W m^−2^), the bistable structure persists, but the barrier protecting the ice-covered state is reduced and the ice-free well becomes deeper. **d** At the second tipping point (Δ*F* ≈ 12.87 W m^−2^), the ice-covered valley vanishes entirely, leaving only the ice-free state.

To explore how Arctic stability evolves under long-term climate change, we vary the external radiative forcing anomaly, Δ*F*. Physically, Δ*F* represents the net additional energy trapped in the Arctic as an effective increase in incoming long-wave radiation (Methods). The resulting bifurcation structure (Fig. 2) provides a structural overview of the system’s response to forcing. At low or negative forcing, the perennial ice-covered state (blue) is the only stable solution and is deeply resilient. As forcing rises, the ice-covered branch approaches the unstable ridge (dashed) separating it from the ice-free state (red). At the upper critical threshold ($\Delta {F}_{2}^{* }\approx +12.87\,{\rm{W}}\,{{\rm{m}}}^{-2}$) the two conditions merge in a saddle-node bifurcation (Fig. 3), eliminating the ice-covered attractor and triggering an abrupt transition to the summer ice-free regime. Crucially, this transition is not easily reversible. The reverse path is asymmetric: perennial ice re-appears only under substantial cooling ($\Delta {F}_{1}^{* }\approx -37.37\,{\rm{W}}\,{{\rm{m}}}^{-2}$), producing pronounced hysteresis. This asymmetry implies that once summer ice is lost, stabilizing forcing at today’s levels, or even returning to preindustrial values, will be insufficient to restore the former perennial state. Instead, substantial additional cooling would be required to push the system back across the stability barrier into the deep ice-covered well. The quoted bifurcation values and barrier heights correspond to the baseline parameter set (Supplementary Table 1). A sensitivity analysis over physically plausible ranges of Δ*E*, albedo contrast, *B*, and *S* (Supplementary Fig. 5) shows that the qualitative bistable structure and hysteresis are robust, whereas the numerical values of ΔF^*^ and Δ*U*/*σ*^2^ depend on parameter choice. Among the parameters tested, albedo contrast (*α*_ice_ − *α*_ocean_) has the strongest effect: a larger contrast widens the bistable range (notably by raising $\Delta {F}_{2}^{* }$) and substantially increases Δ*U*/*σ*^2^, making the ice-covered state more resilient to stochastic perturbations. By contrast, a smoother ice-ocean transition (larger Δ*E*) or stronger longwave damping (larger *B*) narrows the hysteresis loop and reduces the barrier height; higher effective shortwave *S* shifts both tipping points to more negative forcing while increasing Δ*U*/*σ*^2^ within the bistable regime.

The resilience potential provides a dynamic view of how stability changes as forcing rises (Fig. 3; Supplementary Fig. 4). At the lower tipping point (Δ*F* ≈ − 37.37 W m^−2^; Fig. 3a), the system is monostable, with a single deep ice-covered minimum. At intermediate negative forcing (Δ*F* = − 12.25 W m^−2^; Fig. 3b), the system is bistable, with ice-covered and ice-free local minima separated by an unstable ridge. At present-day forcing (Δ*F* = 0 W m^−2^; Fig. 3c), the bistable structure persists, but the barrier protecting the ice-covered state is reduced and the ice-free well becomes deeper. As forcing approaches the upper tipping point (Δ*F* ≈ 12.87 W m^−2^; Fig. 3d), the barrier collapses, the ice-covered and unstable branches merge, and only the ice-free attractor remains.

**Fig. 4:**
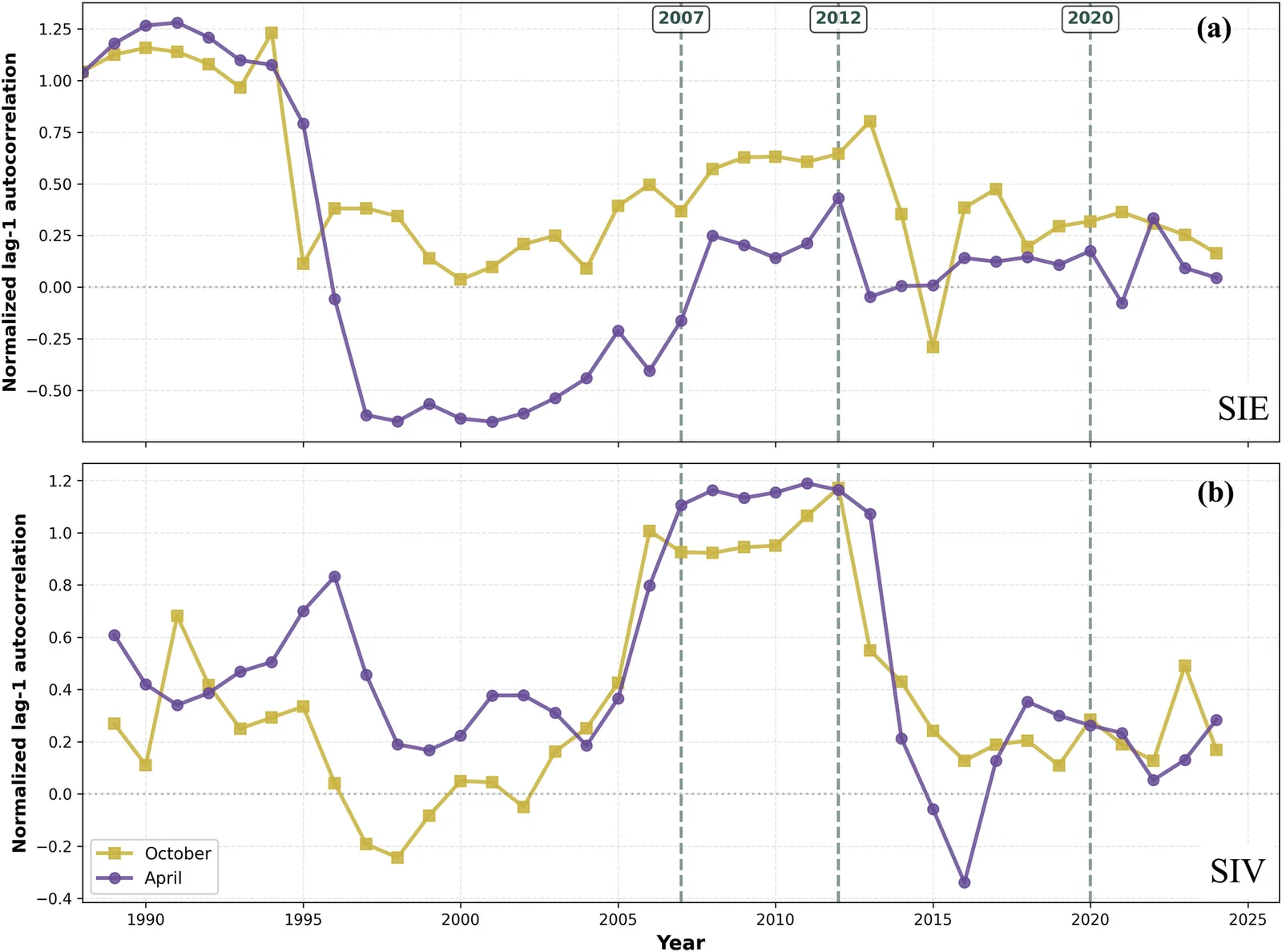
Contextual recovery diagnostics from lag-1 autocorrelation. Lag-1 autocorrelation (AC1) is shown as a contextual local-recovery diagnostic for (**a**) Sea ice extent (SIE) and (**b**) Sea ice volume (SIV). Rolling AC1 is computed from annual month-specific series for April (purple) and October (gold) using a 10-yr window for SIE and an 11-yr window for SIV, after removing a low-order trend within each window. Dashed vertical lines mark 2007, 2012, and 2020.

**Fig. 5:**
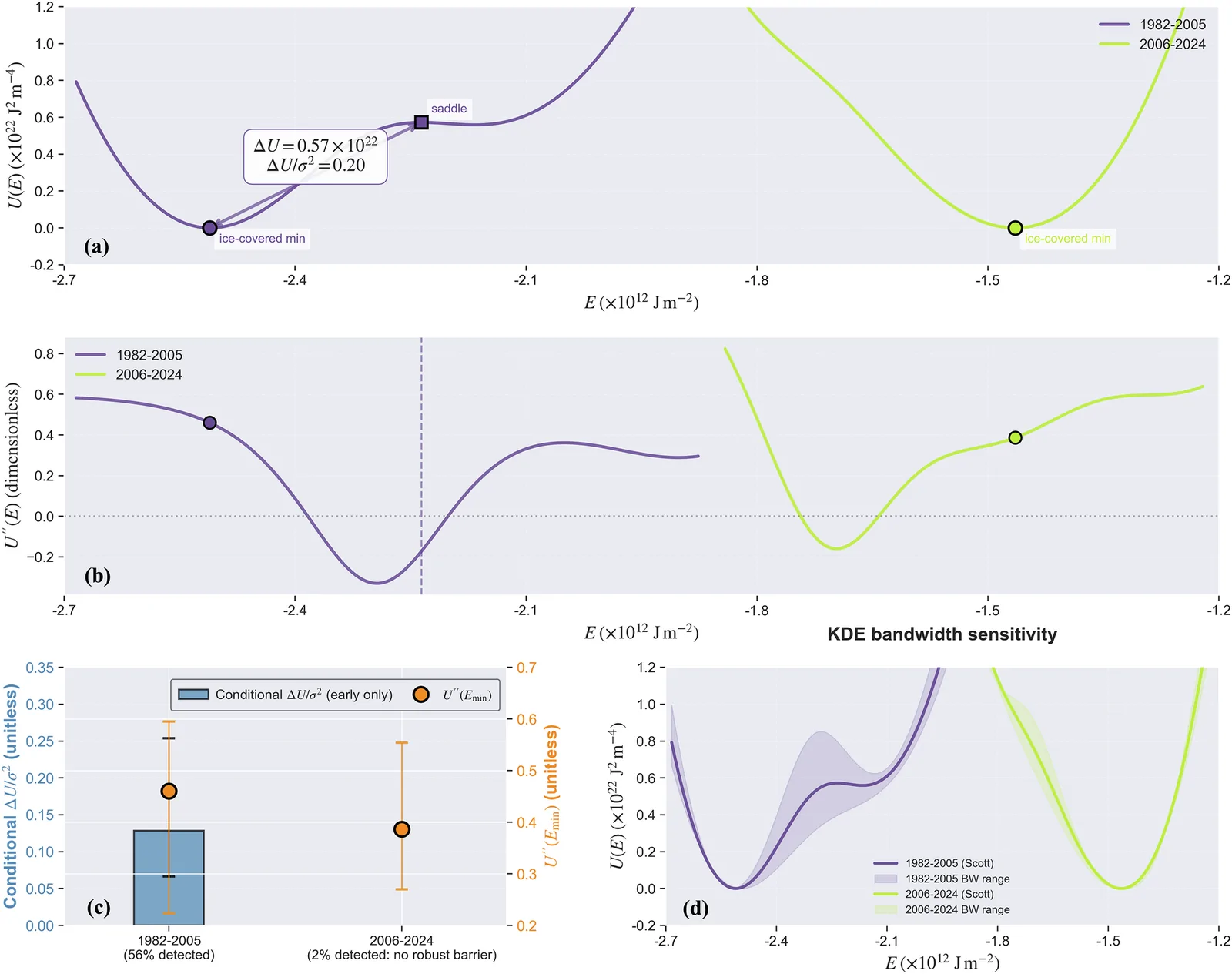
Empirical potential reconstruction from October sea ice volume-derived energy states. **a** Empirical resilience potential *U*(*E*) estimated from October energy *E* = − *κV*/*A*_ref_ in two epochs (1982–2005, purple; 2006–2024, green). Circles indicate the primary ice-covered minima, and the square marks the identifiable interior saddle in the early epoch. Where an interior saddle is robustly identifiable within the sampled range, the barrier from the ice-covered minimum to that saddle defines Δ*U*. **b** Curvature *U''*(*E*) for the same epochs; curvature is interpreted here as a local stability diagnostic only at stationary points. **c** Bootstrap summary metrics by epoch. The conditional barrier-to-noise proxy Δ*U*/*σ*^2^ is plotted only for the early epoch, where an empirical barrier is detected in 56% of bootstrap realizations. For the recent epoch, an identifiable barrier is recovered in only 2% of realizations; therefore, the recent Δ*U*/*σ*^2^ is not plotted as a comparable physical metric and is labeled as having no robust barrier. Orange points show ${U}^{{\prime\prime} }({E}_{\min })$ at the primary empirical minimum, with uncertainty ranges estimated from the same bootstrap procedure. Noise estimates *σ*_*E*_ used in the normalization are reported in Supplementary Table 2. **d** Kernel-bandwidth sensitivity showing how the inferred potential geometry varies across bandwidth choices for both epochs. The shaded ranges indicate bandwidth sensitivity around the Scott bandwidth estimate. This panel is used as a robustness check on empirical potential geometry rather than to define a recent-period barrier when the bootstrap detection frequency is low.

The evolution of the potential in the reduced energy balance model provides one physical interpretation of how Arctic stability may weaken under increasing forcing. In observations, however, alternative explanations remain plausible, including a single drifting basin with changing variance and memory. We therefore do not treat potential-well behavior as established a priori. Instead, in the following section, we compare contextual diagnostics of recovery and variability (Fig. 4 and Supplementary Figs. S9, S10) with empirical potential analogs inferred from October sea ice volume-derived energy states (Fig. 5), and assess whether a barrier-based interpretation is robustly identifiable in the early and recent records.

### Observed recovery diagnostics and limits of empirical barrier inference

We examine contextual local-recovery behavior using lag-1 autocorrelation (AC1) of SIE and SIV (Fig. 4) in April and October. These two metrics measure complementary dimensions of Arctic sea ice: SIE reflects horizontal coverage constrained by geometry, while SIV integrates thermodynamic and dynamic memory through ice thickness. Neither SIE nor SIV exhibits a sustained positive trend in AC1 over the full 1979–2024 record. The clearest signal is a localized enhancement in SIV AC1 during the interval leading into the 2007-2012 extreme minima, after which both seasonal series decline again, more clearly in April than in October (Fig. 4b). SIE AC1 (Fig. 4a) varies more weakly and shows no comparably clear long-term change. We therefore do not use AC1 as evidence for sustained resilience loss or proximity to a tipping point. Instead, we treat it as a contextual local-recovery diagnostic whose variability may reflect transition structure, changing thickness memory, or evolving forcing, in addition to any change in restoring rate. The underlying annual April/October series and their rolling variances are now shown in Supplementary Figs. S9, S10 to make this interpretation transparent. Because SIV integrates thickness memory more directly than SIE, the empirical potential analysis below focuses primarily on October SIV-derived energy states rather than on AC1 alone.

Empirical resilience potentials inferred from October SIV-derived energy states (Fig. 5) provide an observation-based test of the potential framework. Between the early epoch (1982-2005) and the recent epoch (2006-2024), the occupied ice-covered state shifts toward less negative energy, consistent with reduced October ice volume-derived latent energy storage. In the epoch reconstruction shown in Fig. 5a, the early epoch contains an identifiable interior saddle, yielding Δ*U* ≈ 0.57 × 10^22^ J m^−2^ and Δ*U*/*σ*^2^ ≈ 0.20. By contrast, the recent-epoch reconstruction is dominated by a single occupied empirical well within the sampled range, so a recent-period barrier height is not defined as a comparable physical metric. This distinction is important: the observed shift in the occupied state indicates a change in the sea ice energy state, whereas Δ*U* and Δ*U*/*σ*^2^ are conditional barrier diagnostics that are reported only when a local minimum and interior saddle are robustly identified. The curvature at the primary empirical minimum changes only modestly and with overlapping confidence intervals, indicating no clearly resolved change in local linear recovery.

**Fig. 6:**
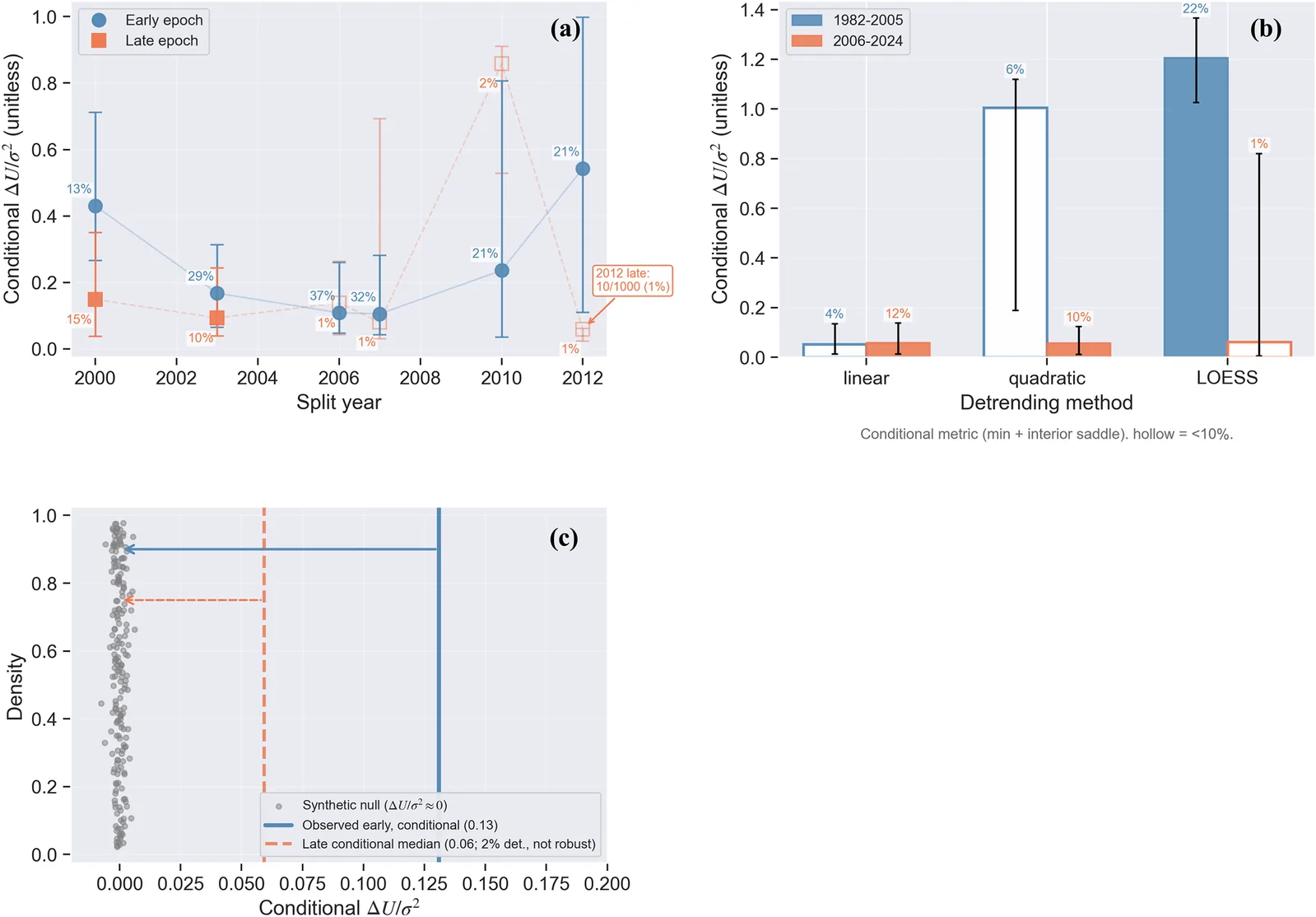
Robustness tests for the conditional empirical barrier diagnostic. **a** Epoch-split sensitivity of the conditional barrier metric Δ*U*/*σ*^2^ for early and late epochs, using alternative split years (2000, 2003, 2006, 2007, 2010, 2012). Points show bootstrap medians and error bars show 68% intervals, computed only from bootstrap realizations in which an empirical barrier, defined by a local minimum and interior saddle, is identified. Percent labels give the identifiable-barrier fraction, and hollow symbols indicate detection fractions below 10%. For the 2012 split, the late-epoch fraction is explicitly annotated as 10/1000 (1%) because narrow uncertainty intervals can reflect very few valid barrier-detected bootstrap draws rather than a well-constrained physical estimate. **b** Detrending sensitivity of the conditional barrier metric Δ*U*/*σ*^2^ to the choice of local detrending within each analysis window (linear, quadratic, LOESS). Bar heights and uncertainty ranges are conditional on successful barrier identification. Values with low detection fractions, shown as hollow bars, should be interpreted as preprocessing-sensitivity outcomes rather than robust physical barrier estimates. **c** Synthetic controls and observed conditional barrier metric. Gray points show Δ*U*/*σ*^2^ from AR(1)+drift monostable synthetic series when an apparent barrier is identified. Vertical lines indicate observationally inferred conditional medians for the default early and late epochs; the late-epoch median is shown only as a low-detection conditional value and is labeled as not robust. The figure is used as a sensitivity and robustness test of the empirical-potential pipeline and to identify the limits of barrier-based interpretation.

Bootstrap resampling quantifies this difference in empirical potential geometry. Under the barrier-identification criterion used here, an identifiable barrier is recovered in 56% of early-epoch realizations but only 2% of recent-epoch realizations. Equivalently, 98% of recent-epoch bootstrap realizations are reconstructed without an identifiable empirical barrier. We therefore do not interpret the rare recent-epoch barrier detections as evidence for a weaker but meaningful recent barrier. Instead, the recent epoch is most consistently represented as a single empirical well under the adopted preprocessing and density-estimation choices. Consequently, Fig. 5c reports the conditional Δ*U*/*σ*^2^ diagnostic only for the early epoch and labels the recent epoch as having no robust barrier. The rare recent-epoch realizations in which a second feature appears are treated as sensitivity outcomes. Kernel-bandwidth sensitivity tests (Fig. 5d) and robustness analyses (Fig. 6) show that the inferred empirical potential geometry depends on bandwidth, detrending choice, epoch definition, and barrier-identification criteria. Across these tests, the most robust observational result is the shift in the occupied energy state, together with limited support for a distinct recent-period empirical barrier. This observational result constrains how the reduced stochastic model should be interpreted. Within the model, when a well-defined barrier exists, shorter mean escape times are obtained for smaller Δ*U*/*σ*^2^, and this qualitative dependence remains when the forcing is changed from white noise to short-correlated Ornstein-Uhlenbeck noise (Supplementary Fig. 6). However, because the recent empirical reconstruction does not robustly identify a barrier, we do not apply this escape-time interpretation quantitatively to the recent observational epoch.

Taken together, the observational diagnostics do not provide uniform evidence for monotonic resilience loss or for a well-defined recent-period empirical barrier. AC1 is best interpreted as a contextual local-recovery diagnostic, whereas the October empirical-potential reconstruction indicates a shift of the occupied ice-covered state toward reduced latent energy storage, while showing that a recent-period empirical barrier is not robustly identifiable. We therefore interpret the observational analysis as supporting a changing occupied sea ice energy state, while constraining the reduced potential framework: the recent epoch should not be treated as a robust double-well system or as evidence for a systematic reduction in the conditional barrier diagnostic. A comparison with CMIP5 model output^2^ shows that the observed 1979-2011 September SIE trend lies within the spread of the CMIP5 trends reported by ref. ^2^, close to the ensemble median shown in Supplementary Fig. 2. This comparison is used only as a scope check confirming that the observed decline has the same sign and comparable magnitude as the model ensemble.

### Robustness and alternative hypotheses

The empirical potential reconstruction (Method) depends on methodological choices because it assumes quasi-stationarity within each analysis window, removes slow forced drift through detrending, and estimates probability densities using kernel density estimation (KDE) for a bounded climate variable. We therefore conduct three robustness and alternative-hypothesis tests to quantify how the inferred potential geometry and barrier metric Δ*U*/*σ*^2^ depend on (i) the definition of early/late epochs and window length, (ii) the detrending method used within each window, and (iii) the possibility that barrier-like features arise in bounded, drifting monostable dynamics. These tests do not establish deterministic bistability; instead, they evaluate how sensitive the conditional barrier diagnostic is to reasonable alternative assumptions and identify the conditions under which barrier-based interpretations are supported by the empirical reconstruction.

The period separation, early (1982–2005) and late (2006–2024) period, was motivated by the transition into the post-2007 interval characterized by recurrent extreme summer minima and a persistently thinner ice pack; however, we emphasize that no single split year is unique. We therefore repeat the empirical potential inference across alternative early/late splits (2000, 2003, 2006, 2007, 2010, and 2012) and in moving 20-year windows (Fig. 6a). This sensitivity test is used to determine when the conditional barrier diagnostic is stable under reasonable changes in epoch definition. Across the tested split years, the inferred early-late ordering of Δ*U*/*σ*^2^ is not invariant: for some split years the late-epoch conditional value is lower than the early-epoch value, whereas for other split years this ordering reverses. Because Δ*U*/*σ*^2^ is defined only for bootstrap realizations in which an empirical barrier is identified, these conditional values must be interpreted together with the corresponding barrier-detection fractions. In low-detection cases, narrow bootstrap intervals may reflect the small number of valid barrier-detected realizations rather than a well-constrained physical estimate. We therefore interpret Fig. 6a as a robustness test that delineates the limits of a barrier-based interpretation.

Next, KDE-based potentials can be biased by trending or bounded time series, potentially producing spurious shoulders or shallow minima. To test sensitivity to the treatment of forced drift, we repeat the empirical potential reconstruction using multiple detrending methods: linear, quadratic, and LOESS (Fig. 6b). The default preprocessing case used for Fig. 5c is included in Fig. 6b as the matching reference case, while the remaining bars show alternative detrending choices. Across detrending choices, the inferred conditional barrier metric and its uncertainty vary substantially across preprocessing choices. Because Δ*U*/*σ*^2^ is defined only for bootstrap realizations in which an empirical barrier is identified, these values are interpreted together with the corresponding barrier-detection fractions. Fig. 6b, therefore, helps identify when a barrier-based interpretation is supported by the empirical reconstruction.

To assess whether barrier-like features could arise in a monostable system with forced drift and bounded variability, we construct synthetic control series designed to match key low-order properties of the observations. Specifically, we simulate monostable first-order autoregressive AR(1) processes with a drifting mean (representing forced decline) and innovation variance matched to the observed series; we also test a variant with state-dependent variance to mimic changing variability under thinning ice. We then apply the identical processing chain used for the observations (windowing, detrending, KDE bandwidth selection, and potential reconstruction, ${U}_{{\rm{emp}}}\propto -\log p$) and compute Δ*U*/*σ*^2^ using the same barrier-identification criterion as above. In these monostable controls, identifiable barriers occur only infrequently; when present, they are typically weaker (smaller Δ*U*/*σ*^2^) than the early-period observational reconstructions (Fig. 6c). This indicates that the early-period barrier signal is not a trivial by-product of boundedness plus drift under the tested null models. Nevertheless, this null-hypothesis test is not exhaustive: more complex non-Gaussian, multi-timescale, or strongly state-dependent monostable processes could in principle generate barrier-like features. We therefore use the synthetic controls as a null-model test of the empirical-potential pipeline: they indicate that early-epoch barrier-like features are not a trivial artefact of the simple monostable controls considered here, while the recent-period interpretation is constrained by the separate barrier-detection and robustness analyses above.

### Stochastic forcing and noise-induced tipping

In the reduced stochastic model, potential flattening implies slower local recovery near the occupied minimum, while a lower model-defined barrier increases susceptibility to noise-induced excursions. In the real Arctic, such perturbations are pervasive: synoptic storms, warm-air intrusions, Atlantic Water inflows, and cloud–radiation anomalies continually disturb the surface energy balance. Incorporating this stochastic forcing is therefore essential to quantify not only the mean stability but also the probability and timing of transitions. To capture these effects, we extend the energy balance model with a Langevin formulation (Methods), in which the system evolves on its resilience potential while being continually perturbed by random noise. This stochastic extension links observed short-term variability directly to fluctuations in the underlying energy budget and provides a framework for interpreting noise-induced transitions alongside deterministic forcing. Within the reduced model, flattening of the potential weakens the restoring force near the occupied minimum and shortens the expected escape time from the ice-covered state. In observations, however, AC1 and variance can at most provide contextual information about local recovery and variability; they do not by themselves diagnose barrier height or prove bistability. We therefore use the stochastic extension primarily to interpret model behavior and to relate local recovery diagnostics to conditional barrier metrics: local proxies such as AC1, variance, and curvature near a minimum concern recovery around the occupied state, whereas Δ*U* and Δ*U*/*σ*^2^ are meaningful only when a well-defined barrier exists.

**Fig. 7:**
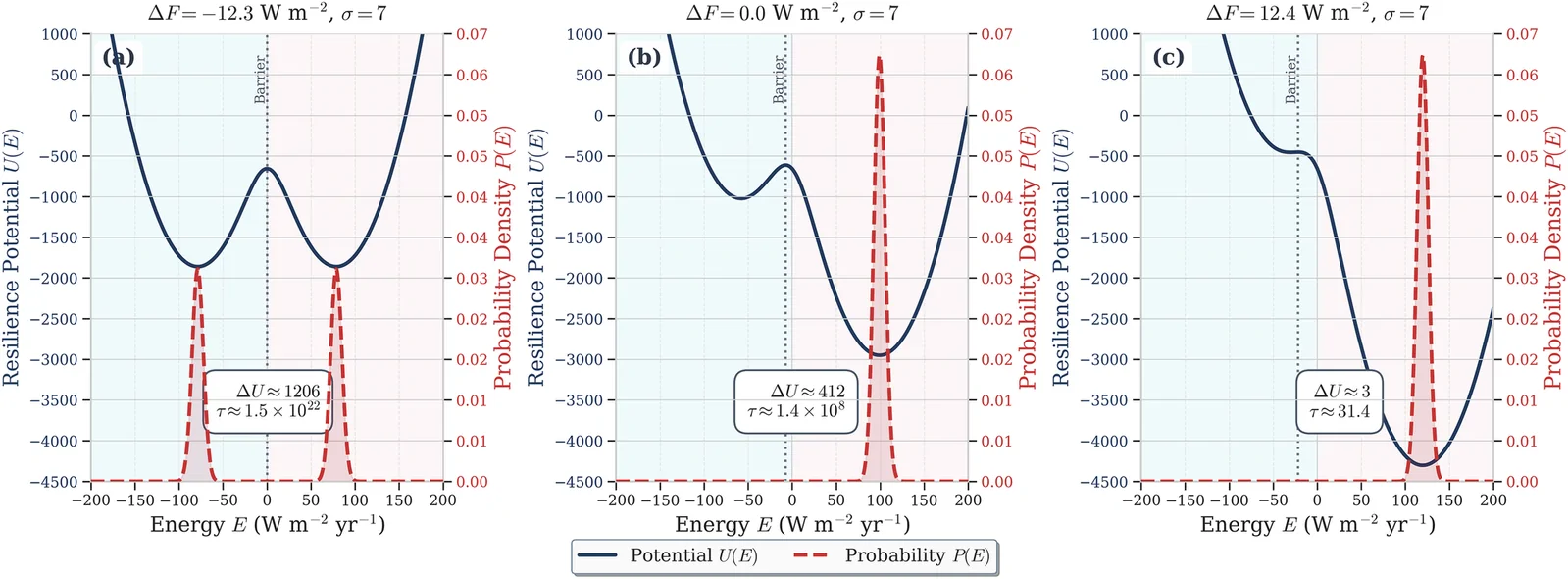
The resilience potential *U*(*E*) (black) and steady-state probability density *P*(*E*) (red) are shown for three representative forcing levels in the reduced stochastic energy-balance model. **a** In an ice-favored bistable regime (Δ*F* ≈ − 12.3 W m^−2^), the model-defined potential barrier is high (Δ*U* ≈ 1206), yielding a sharply peaked bimodal distribution and an extremely long modeled escape time (*τ* ≈ 1.5 × 10^22^). **b** Under the baseline forcing case (Δ*F* = 0 W m^−2^), the system remains bistable but the model-defined barrier is substantially reduced (Δ*U* ≈ 412), so modeled escape times shorten (*τ* ≈ 1.4 × 10^8^) and the ice-free well becomes more probable. **c** Near the upper tipping threshold, the plotted case uses Δ*F* ≈ 12.4 W m^−2^, approximately 0.5 W m^−2^ below the model bifurcation value $\Delta {F}_{2}^{* }\approx 12.87\,{\rm{W}}\,{{\rm{m}}}^{-2}$. In this near-threshold case, the model-defined barrier nearly collapses (Δ*U* ≈ 3), *P*(*E*) shifts strongly toward the ice-free attractor, and the modeled escape time is reduced to *τ* ≈ 31.4. These representative panels illustrate how the model-defined barrier and modeled escape time decrease as forcing approaches the upper bifurcation point; they are not used as direct observational evidence for a recent empirical barrier.

Model experiments illustrate this progression (Fig. 7). In an ice-favored bistable regime (*ΔF* ≈ − 12.3 W m^−2^; Fig. 7a), the model-defined barrier is high (Δ*U* ≈ 1206), yielding an extremely long modeled escape time (*τ* ≈ 1.5 × 10^22^). Under the baseline forcing case (Δ*F* = 0 W m^−2^; Fig. 7b), the system remains bistable, but the model-defined barrier is substantially reduced (Δ*U* ≈ 412), so modeled escape times shorten (*τ* ≈ 1.4 × 10^8^) and the ice-free well becomes more probable. Near the upper tipping threshold, the plotted case in Fig. 7c uses Δ*F* ≈ 12.4 W m^−2^, approximately 0.5 W m^−2^ below the model bifurcation value $\Delta {F}_{2}^{* }\approx 12.87\,{\rm{W}}\,{{\rm{m}}}^{-2}$. In this near-threshold case, the model-defined barrier nearly collapses (Δ*U* ≈ 3), the stationary probability density shifts strongly toward the ice-free attractor, and the modeled escape time is reduced to *τ* ≈ 31.4. The forcing Δ*F* in this reduced model is an effective net energy flux and is not directly comparable to top-of-atmosphere radiative forcing.

This stochastic perspective also clarifies recent abrupt melt events. The 2012 record minimum, preconditioned by early melt pond formation^61^ and multiple warm air intrusions^66^, and the 2020 minimum, associated with persistent clear skies and amplified ocean-atmosphere coupling over the Siberian shelf^67^, both illustrate how transient anomalies can produce disproportionately large late-season ice losses when the background state is already weakened. In this sense, the reduced stochastic framework provides a model-based interpretation of enhanced susceptibility to large late-season ice losses under a weakened background state, while leaving the existence of fully resolved observational inter-well transitions to be assessed by the empirical potential diagnostics.^25,68^.

## Discussion

Although Arctic sea ice decline is commonly interpreted as a largely quasi-linear forced response to greenhouse warming^10,32,33^, that view does not preclude changes in the stability properties of the declining ice cover itself. Here, we show that a resilience potential framework offers a useful complementary lens for examining how persistence, local recovery, occupied-state shifts, and conditional barrier diagnostics can be linked in a reduced Arctic sea ice model. We therefore interpret the empirical results as an observation-based constraint on potential-based resilience concepts, supporting a changing occupied sea ice energy state while limiting claims of contemporary bistability or systematic weakening of a recent-period barrier^20,69^.

Unlike idealized conceptual models, our formulation is constrained by satellite-derived radiative fluxes and empirically observed albedo energy feedbacks, enabling resilience diagnostics to be evaluated against multi-sensor records^20,63^. In the reduced stochastic model, incorporating stochastic forcing shows that the persistence of the ice-covered state depends sensitively on the model-defined potential barrier: under the baseline parameter set, this state remains metastable on long timescales, but as forcing approaches the upper tipping threshold (Δ*F* ≈ 12.87 W m^−2^), the barrier is substantially lowered and modeled escape times shorten dramatically. In such low-barrier model regimes, individual synoptic or oceanic anomalies can trigger disproportionate ice losses, consistent with record-low sea ice minima in 2007, 2012, and 2020, when transient atmospheric and oceanic circulation patterns may have produced disproportionately large ice losses in an already fragile system^33,66,70^.

The reduced energy balance model also exhibits pronounced hysteresis. Once the ice-covered state collapses, simply returning radiative forcing to present-day or even pre-industrial levels would be insufficient to restore perennial ice. Recovery would instead require sustained cooling and is likely to unfold over centuries, Consistent with previous modeling studies^19,71^. Hysteresis in this sense reflects a distinction between vulnerability and reversibility: the system may become more susceptible to large perturbations as the protective barrier weakens, yet recovery to an earlier state can still remain difficult. This separation between resistance and reversibility provides a useful lens for interpreting how Arctic sea ice stability changes as the climate warms.

More broadly, the framework developed here provides a pathway for diagnosing resilience across other climatic tipping elements, including the Greenland Ice Sheet, the Atlantic meridional overturning circulation, and the Amazon rainforest, by quantifying how deterministic forcing and stochastic variability jointly shape stability^72^. Destablization of Arctic sea ice may further interact with these subsystems through albedo and circulation feedbacks, raising the risk of cascading transitions within the Earth system^19,25,73^.

Looking ahead, future progress will require extending this approach, including the combined use of CERES, CryoSat-2, and ICESat-2 observations to evaluate empirical potential diagnostics with independent radiative, thickness, and freeboard constraints^12^, embedding the diagnostic within Earth System Models to assess abrupt-change likelihood, and integrating data assimilation and machine-learning emulators for near-real-time monitoring and early warning. Taken together, our results show that the reduced stochastic EBM provides a useful framework for linking model-defined stability barriers, local recovery diagnostics, and observation-derived shifts in the Arctic sea ice energy state. The current empirical reconstruction supports a changing occupied ice-covered state while constraining how far a recent-period barrier interpretation can be taken. Future work should therefore test these empirical potential diagnostics using longer records, spatially resolved thickness observations, Earth system model ensembles, and alternative stochastic-process null models.

## Methods

We use a continuous energy-balance model to represent the coupled evolution of Arctic sea ice and the upper ocean. The model unifies ice-covered (*E* < 0) and open-water (*E* > 0) conditions under a single state variable, *E*, describing the net surface energy of the system. Stability is quantified through a resilience potential, *U*(*E*), derived from a smooth albedo-energy feedback *α*(*E*) that captures the gradual transition from bright ice to dark open water. By systematically varying external radiative forcing (Δ*F*), expressed as an effective increase in incoming long-wave radiation, we map how the Arctic system evolves between coexisting ice-covered and ice-free equilibria, identifying the thresholds, hysteresis, and metastable behavior shown in Fig. 2 and Supplementary Figs. 1–4.

### Core energy-balance model (Equations 1–6)

We first group the model definition, potential formulation, and local stability diagnostics in a compact form.

#### Albedo-energy feedback

The key nonlinearity is the albedo-energy feedback, represented by a smooth sigmoidal transition between ice-covered and open-water albedos:

1 $$\alpha (E)\,=\,{\alpha }_{{\rm{ocean}}}\,+\,\frac{{\alpha }_{{\rm{ice}}}-{\alpha }_{{\rm{ocean}}}}{1+{e}^{E/\Delta E}}.$$

Here *α*_ice_ = 0.7 and *α*_ocean_ = 0.07 represent characteristic albedos, and Δ*E* controls the sharpness of the transition.

#### Basin-mean energy balance

The evolution of the basin-mean energy state *E* is governed by absorbed shortwave minus linearized outgoing longwave radiation:

2 $$\frac{dE}{dt}=\mathop{\underbrace{[1-\alpha (E)]\,S}}\limits_{{E}_{{\rm{in}}}}\,-\,\mathop{\underbrace{(A+BE-\Delta F)}}\limits_{{E}_{{\rm{out}}}}\,.$$

Steady states satisfy *E*_in_(*E*) = *E*_out_(*E*) (Supplementary Fig. 3).

#### Potential definition

We define a resilience potential *U*(*E*) such that the deterministic dynamics follow the negative gradient of *U*:

3 $$\frac{dE}{dt}\,=\,-\,\frac{\partial U}{\partial E}\,.$$

#### Closed-form potential

Integrating Eq. (2) yields:

4 $$U(E)=[A-\Delta F-S(1-{\alpha }_{\mathrm{ocean}})]\,E+\frac{B{E}^{2}}{2}+S({\alpha }_{\mathrm{ice}}-{\alpha }_{\mathrm{ocean}})\left\{E-\Delta E\,\mathrm{ln}\,\left(1+{e}^{E/\Delta E}\right)\right\}+C,$$

where *C* is an integration constant (set to zero).

#### Local stability from curvature

Local stability is diagnosed from the curvature:

5 $$\frac{{d}^{2}U}{d{E}^{2}}=B-S\frac{{\alpha }_{\mathrm{ice}}-{\alpha }_{\mathrm{ocean}}}{\Delta E}\,\frac{{e}^{E/\Delta E}}{{(1+{e}^{E/\Delta E})}^{2}}\,\left\{\begin{array}{cc} > 0, & \mathrm{stable}\,\mathrm{minimum},\\ < 0, & \mathrm{unstable}\,\mathrm{maximum}.\end{array}\right.$$

#### Stochastic extension

To account for high-frequency atmospheric/oceanic variability, we extend Eq. (3) to a Langevin equation:

6 $$\frac{dE}{dt}\,=\,-\,\frac{\partial U}{\partial E}\,+\,\sigma \,\xi (t),$$

where *ξ*(*t*) is Gaussian white noise with $\langle \xi (t)\,\xi ({t}^{{\prime} })\rangle =\delta (t-{t}^{{\prime} })$ and *σ* is the noise amplitude (estimated from observations below; Supplementary Fig. 6 explores colored-noise sensitivity).

### Albedo-energy feedback

Unlike earlier stepwise parameterizations, Eq. (1) provides a continuous transition that is both physically motivated (gradual darkening via thinning ice, snow metamorphism, and melt ponds) and mathematically tractable. The associated albedo curve is shown in Supplementary Fig. 1.

To place the basin-mean diagnostics in context, we compare the sign and order of magnitude of observed trends to published multi-model results. Following^2^, we take the 1979–2011 September sea ice extent trend (in 10^6^km^2^ per decade) from the same observational record used in our resilience pipeline and compare it to the distribution of 1979–2011 trends from 20 CMIP5 models reported in that study (Supplementary Fig. 2). This provides a consistency check without attempting full validation of the reduced model against spatially resolved simulations; the comparison is for scope only, to ensure that aggregate observed behavior lies in a plausible range relative to current-generation climate models.

### Arctic sea ice energy balance model

In the ice-covered regime (*E* < 0), *E* represents latent heat stored in the ice pack and scales with mean thickness; in the ice-free regime (*E* > 0), *E* represents sensible heat in the mixed layer. This unified formulation avoids discontinuities at the ice-ocean transition and enables a single stability landscape across regimes.

### Resilience potential formulation

This reformulation provides an intuitive resilience potential in which local minima represent stable equilibria and local maxima represent unstable thresholds (Supplementary Fig. 4). In this context, the curvature *U*^*″*^(*E*) is interpreted as a local stability diagnostic only at stationary points, where the first derivative vanishes. At a minimum, larger positive curvature indicates faster local recovery from perturbations; at a maximum, negative curvature indicates local instability. Away from stationary points, curvature alone is not a sufficient stand-alone measure of stability.

### Forcing and bifurcation analysis

We analyze how steady states and the potential change with external forcing Δ*F*. The forcing was varied systematically to construct bifurcation diagrams and identify two critical thresholds. At $\Delta {F}_{2}^{* }\approx +12.87\,{\rm{W}}\,{{\rm{m}}}^{-2}$ the ice-covered attractor merges with the unstable state and disappears in a saddle-node bifurcation, forcing an abrupt transition to the ice-free state; upon reducing the forcing, the system does not return to the ice-covered branch until Δ*F* is lowered to a much smaller value, $\Delta {F}_{1}^{* }\approx -37.37\,{\rm{W}}\,{{\rm{m}}}^{-2}$, implying substantial hysteresis in this reduced model. We emphasize that these thresholds are properties of the idealized basin-mean energy balance and should not be interpreted as literal top-of-atmosphere forcing values in comprehensive climate models. Bifurcation diagrams (Fig. 2) were constructed numerically by sweeping ΔF over a prescribed range and solving *E*_in_(*E*) = *E*_out_(*E*) for all intersections using a root-finding routine. Stability of each intersection was determined from the sign of *d*(*dE*/*dt*)/*dE* (equivalently *U*^*″*^(*E*) from Eq. (5)).

### Parameterization and sensitivity

The baseline parameters (*A*, *B*, *S*, *α*_ice_, *α*_ocean_, Δ*E*) are chosen so that the linearized outgoing longwave term *A* + *BE* and the absorbed shortwave term  [1-α(E)]S reproduce the sign and approximate magnitude of basin-mean Arctic net radiative damping and seasonal shortwave forcing, while Δ*E* controls the smoothness of the ice-ocean transition in the sigmoid albedo function. Because the model is conceptual, we interpret the resulting thresholds $\Delta {F}_{1,2}^{* }$ as effective forcings within a reduced energy balance rather than as literal top-of-atmosphere radiative perturbations. To assess robustness, we vary Δ*E*, the albedo contrast *α*_ice_ − *α*_ocean_, *B*, and *S* over physically plausible basin-mean ranges (absolute values; see Supplementary Table 1) and recompute (i) the existence of multiple equilibria, (ii) the barrier height Δ*U*, and (iii) the barrier-to-noise proxy Δ*U*/*σ*^2^. The sensitivity of $\Delta {F}_{1}^{* }$, $\Delta {F}_{2}^{* }$, and Δ*U*/*σ*^2^ to each parameter is shown in Supplementary Fig. 5. The sensitivity ranges in Supplementary Table 1 were chosen as physically plausible basin-mean perturbations around the baseline parameter set, intended to span meaningful weakening-to-strengthening of each feedback or damping term while preserving an interpretable Arctic radiative-balance regime in the reduced model; they are therefore not formal observational uncertainty bounds. The qualitative emergence of a metastable regime with coexisting ice-covered and ice-free attractors is robust across this parameter space, whereas the precise values of ΔF^*^ and the associated escape times are parameter dependent.

### Stochastic extension

Equation (6) includes stochastic perturbations representing synoptic weather systems, warm-air intrusions, and anomalous ocean heat pulses. The noise amplitude *σ* is estimated directly from the observed October energy series *E*_obs_ within each analysis window as the AR(1) innovation scale, *σ*^2^ = Var(*E*_obs_) (1 − *ϕ*^2^), where *ϕ* is the lag-1 autoregressive coefficient from the AR(1) fit (Methods: Empirical October potential), and these observationally constrained values are used in the barrier-to-noise ratio Δ*U*/*σ*^2^ and in Kramers-type escape-time scaling. Published basin-mean estimates are used only as a consistency check.

For October energy in our two analysis epochs this procedure yields *σ*_*E*_ ≈ 1.7 × 10^11^ J m^−2^ (1982-2005) and *σ*_*E*_ ≈ 1.8 × 10^11^ J m^−2^ (2006-2024), i.e., ~ 0.17 − 0.18 × 10^12^ J m^−2^ (Supplementary Table 2), which is consistent in magnitude with previously published basin-mean estimates of order 7 W m^−2^ for the modern Arctic.

To assess sensitivity to temporal persistence, we also repeat the escape time and stationary-density calculations using an Ornstein-Uhlenbeck (red-noise) forcing with decorrelation times of order 1 ~ 4 weeks; the qualitative dependence of the mean escape time *τ* on Δ*U*/*σ*^2^ is unchanged, while persistence reduces effective escape times relative to white-noise estimates for the same marginal variance (Supplementary Fig. 6).

### Mapping observed sea ice extent and sea ice volume to the resilience state

#### Sea ice extent (SIE): preprocessing and contextual diagnostics

Monthly pan-Arctic SIE is ingested as a continuous record from January 1979. We form a signed state variable for resilience analysis,

7 $$X(t)=-\,{\rm{SIE}}(t),$$

so that larger ice cover maps to more negative values. To isolate low-frequency changes in recovery dynamics at the seasonal peaks, we extract one value per year for April (late winter) and October (early refreezing). For each month-specific annual series, we compute variance and lag-1 autocorrelation (AC1) in rolling 10-year windows, after removing a low-order trend within each window. Autocorrelations are Fisher-*z* transformed for display and comparability. Trends in AC1 are evaluated using Kendall’s *τ*, with *p*-values reported alongside (windowed) confidence intervals. Using one datum per year avoids aliasing from intra-annual variability and ensures that the extent-based contextual diagnostics series are aligned to the same year axis as the volume-based series.

#### Sea ice volume (SIV): dataset harmonization, ensemble construction, and contextual diagnostics

Monthly pan-Arctic sea ice volume is compiled from two independent sources: the C3S reconstruction (available from 1979 onward) and the PIOMAS reanalysis. Because the two series exhibit systematic offsets and different scaling over their overlap period, we harmonize PIOMAS to C3S using an ordinary least-squares regression over the overlap (2003–2024), applied separately for April and October (Supplementary Fig. 7 and Supplementary Fig. 8):

8 $${{\rm{PIOMAS}}}_{{\rm{cor}}}=a\cdot {\rm{PIOMAS}}+b,$$

with month-specific coefficients. For April, we obtain *a* = 0.216, *b* = 9053 km^3^, yielding *R*^2^ = 0.26 and *r* = 0.51; the correction removes a mean bias of − 5680 km^3^ and reduces the RMSE from 1171 km^3^ to 486 km^3^. For October, we obtain *a* ≈ 0.26, *b* ≈ 2240 km^3^, with *R*^2^ ≈ 0.58 and *r* ≈ 0.76 (RMSE ≈ 380 km^3^ after correction). Residuals show no discernible temporal trend in either month, confirming the stability of the correction across the overlap years.

Because the regression explains only a modest fraction of monthly variance for April (*R*^2^ = 0.26), the correction is intended to remove mean bias and scaling differences rather than to reproduce month-to-month variability. We therefore assess robustness by repeating all resilience diagnostics using (i) C3S alone (1979–present), (ii) raw PIOMAS alone, and (iii) the bias-corrected ensemble mean; our qualitative conclusions are unchanged across these choices (Supplementary Fig. 7 and Supplementary Fig. 8), with coefficients *a* = 0.216 and *b* = 9053 km^3^. This correction removes a mean bias of − 5680 km^3^ and reduces the RMSE from 1171 km^3^ to 486 km^3^, yielding *R*^2^ = 0.26 and *r* = 0.51. Residuals show no discernible temporal trend, confirming the correction’s stability across the overlap years.

We then form an ensemble mean of C3S and PIOMAS_cor_ over the full 1979–2024 record to obtain a homogenized, bias-corrected SIV time series. For April and October, we extract the annual month-specific means and compute rolling 11-year variance and lag-1 autocorrelation (AC1), consistent with the longer memory of sea ice thickness. AC1 is Fisher-*z* transformed prior to trend estimation, and the resulting time series are tested for monotonic trends using Kendall’s *τ*. These diagnostics provide a physically consistent description of evolving recovery behavior and variability in the ice volume state, comparable to the SIE-based contextual signals.

#### Mapping observed volume to the resilience state

To compare observations with the model’s resilience potential, we map sea ice volume to an energy state variable *E* that measures latent energy per unit area stored in the pack:

9 $$h(t)=\frac{V(t)}{{A}_{{\rm{ref}}}},\,{E}_{{\rm{obs}}}(t)=-\kappa \,h(t),\,\kappa ={\rho }_{i}\,{L}_{i}.$$

with *ρ*_*i*_ = 917 kg m^−3^ and *L*_*i*_ = 3.34 × 10^5^ J kg^−1^. *A*_ref_ is a fixed reference area used to express the energy density (we adopt *A*_ref_ = 6.25 × 10^8^ m^2^, equivalent to a 25 × 25 km cell normalization based on the dataset). The negative sign indicates the energy required to melt the ice column. We neglect late-summer sensible cold content (< 10%), which is small relative to latent storage. This mapping places the observed Arctic state directly in the model’s ice-covered regime (*E* < 0) and permits comparison with the theoretical equilibria and saddle.

#### Empirical October potential from observations

To infer observational analogs of the resilience potential, we reconstruct an empirical potential *U*_emp_(*E*) from the October distribution of *E*_obs_. October is diagnostically important because it samples the system immediately after the melt season, when residual ocean heat and thin autumn ice encode the memory of summer forcing in *E*. Following the steady-state Fokker–Planck solution of the Langevin model, we estimate:

10 $${U}_{{\rm{emp}}}(E)=-\frac{{\sigma }^{2}}{2}\,\,{\rm{ln}}\,P(E)+C$$

where *P*(*E*) is obtained by kernel density estimation (KDE) of deseasonalized, locally detrended *E*_obs_(*t*) within analysis windows, and *σ* is the innovation scale inferred from an AR(1) fit to the same monthly series (*σ*^2^ = Var(*E*) [1 − *ϕ*^2^]). We repeat the analysis for two epochs, 1982-2005 and 2006-2024, identify the primary ice-covered minimum ${E}_{\min }$, and compute the curvature ${U}^{{\prime\prime} }({E}_{\min })$ as a proxy for local linear recovery. When an interior saddle to the right of ${E}_{\min }$ is robustly identifiable, we also compute the barrier height Δ*U*. Uncertainty is quantified with block-bootstrap resampling over years (typical block length 3 yr; *n* = 10^3^ draws), and robustness is assessed with bandwidth sensitivity (Scott, Silverman, ± 20% of Scott’s factor) and *σ*-method sensitivity (standard deviation, first-order autoregressive residual). Where appropriate, we compare empirical metrics to the analytical potential *U*(*E*) under matched radiative forcing anomalies Δ*F* (see Methods: Resilience Potential formulation). A barrier height Δ*U* is reported only when the reconstructed *U*_emp_(*E*) exhibits two local minima separated by an interior local maximum within the sampled energy range; otherwise, the window is classified as “no identifiable barrier” and Δ*U* is not defined. Previous observational studies have primarily inferred resilience from local stability proxies, such as AC1 or curvature near the occupied state, which quantify the linear recovery rate but do not constrain barrier-crossing behavior. Our empirical potential approach complements these by estimating both (i) local stability via ${U}^{{\prime\prime} }({E}_{\min })$ and (ii) a conditional barrier diagnostic via Δ*U* (and Δ*U*/*σ*^2^), but only when the reconstructed *U*_emp_(*E*) contains an occupied local minimum and a robustly identifiable interior saddle. This diagnostic characterizes one component of stability, because the response to perturbations also depends on the distance from the minimum to the saddle, the width of the well, the curvature near the minimum, the noise structure, and the robustness of the inferred geometry.

#### Statistical indicators and linkage to escape-time theory

For SIE and SIV, we report the evolution of variance and AC1 as contextual diagnostics of local recovery and variability, not as stand-alone evidence of critical slowing down. For the empirical October potential, we summarize changes in ${E}_{\min }$ and ${U}^{{\prime\prime} }({E}_{\min })$, and report Δ*U* and Δ*U*/*σ*^2^ only when a barrier is robustly identifiable. In the stochastic extension of the reduced model, a well-defined barrier maps to mean escape times via Kramers-type scaling,

11 $$\tau \,\propto \,\exp \left(\frac{\Delta U}{{\sigma }^{2}}\right),$$

providing a conceptual bridge between contextual diagnostics (AC1, variance) and model-defined mechanistic diagnostics (curvature and barrier height). We do not apply this scaling quantitatively to empirical windows in which no robust barrier is identified.

### Probability density and escape times

For small *σ*, the steady-state probability density can be approximated^74^ as:

12 $$\begin{array}{l}{P}_{s}(E)={\left\{\sigma \sqrt{\frac{\pi }{{U}^{{\prime\prime} }({E}_{s1})}}\exp (-\frac{2}{{\sigma }^{2}}U({E}_{s1}))+\sigma \sqrt{\frac{\pi }{{U}^{{\prime\prime} }({E}_{s3})}}\exp (-\frac{2}{{\sigma }^{2}}U({E}_{s3}))\right\}}^{-1}\\ \times \exp (-\frac{2}{{\sigma }^{2}}\,U(E))\end{array}$$

This expression shows that for weak noise, the system is most likely to be found near the stable steady states (the minima of the potential *U*(*E*)). As noise increases or the resilience potential flattens, the probability distribution broadens, and the likelihood of finding the system between states (i.e., in transition) increases.

The mean escape time *τ* for the system to transition stochastically out of a potential well over a barrier is given by Kramers’ formula:

13 $$\tau \,\approx \,\frac{2\pi }{\sqrt{| {U}^{{\prime\prime} }({E}_{s2})| \,{U}^{{\prime\prime} }({E}_{s1})}}\,\exp \left(\frac{2| U({E}_{s2})-U({E}_{s1})| }{{\sigma }^{2}}\right).$$

where Δ*U* is the height of the potential barrier, and *U*^*″*^(*E*_*s*1_) and *U*^*″*^(*E*_*s*2_) are the curvatures at the stable state and the barrier top, respectively. In the reduced EBM, where the minimum and saddle are analytically defined, this quantity provides a model-defined barrier diagnostic. In empirical reconstructions, however, Δ*U* is treated as a conditional barrier diagnostic and is reported only when a barrier is robustly identifiable. This equation highlights the crucial exponential sensitivity of modeled escape times to the barrier-to-noise ratio.

### Observational datasets

Satellite observations were used to evaluate the theoretical predictions of the model. Sea ice extent was obtained from the NASA/NSIDC passive microwave data product (1979–2024)^75^. Surface albedo was characterized using the CERES Synoptic TOA and surface fluxes and clouds (SYN) product (2000–2024)^76,77^ together with the longer-term AVHRR narrowband albedo record (1982–2022)^78^. Sea ice volume reconstructions were derived from the Copernicus Climate Change Service (C3S) sea ice thickness product^54^ and the PIOMAS reanalysis^79^. These datasets document concurrent declines in extent, volume, and albedo, and provide the observational basis for the resilience diagnostics examined in this study (Fig. 1).

## Supplementary information

Supplementary Information

## Data Availability

All data used in this study are publicly available from the original data providers. Sea ice extent data are available from NSIDC at https://nsidc.org/data/g02202/versions/4/. CERES products are available from the NASA CERES data portal at https://ceres.larc.nasa.gov/data/. AVHRR albedo data are available from NOAA NCEI at https://www.ncei.noaa.gov/access/metadata/landing-page/bin/iso?id=gov.noaa.ncdc:C01580. Sea ice thickness and volume data are available from the Copernicus Climate Change Service at https://cds.climate.copernicus.eu/datasets/satellite-sea-ice-thickness?tab=documentation and from PIOMAS at https://psc.apl.washington.edu/zhang/IDAO/data_piomas.html.

## Code availability

The code used to process the data and generate the results in this study is publicly available at https://github.com/Lu-Zhou-UU/Arctic_resilience.git.
