# Supplementary material for: Stochastic resilience of Arctic sea ice: a framework bridging theory and observation
Source: NPJ Clim Atmos Sci. 2026 Jun 15;9(1):191. doi: 10.1038/s41612-026-01455-z (PMC13630570; doi:10.1038/s41612-026-01455-z)
Supplement: Supplementary file 1 [file 41612_2026_1455_MOESM1_ESM.pdf]

# Supplementary Information

## Stochastic resilience of Arctic sea ice: a framework bridging theory and observation

Chuixiang Yi[1,2], Lu Zhou[3,*], and Deliang Chen[4,5]

[1]School of Earth and Environmental Sciences, Queens College, City University of New York, New York, NY 11367, USA
[2]Earth and Environmental Sciences Department, Graduate Center, City University of New York, New York, NY 10016, USA
[3]Institute for Marine and Atmospheric Research, Utrecht University, Utrecht 3584 CC, the Netherlands
[4]Department of Earth System Science, Tsinghua University, Beijing 100084, China
[5]Institute for Carbon Neutrality, Tsinghua University, Beijing 100084, China

*Correspondence: l.zhou@uu.nl

This file contains Supplementary Figures 1–10 and Supplementary Tables 1–2.

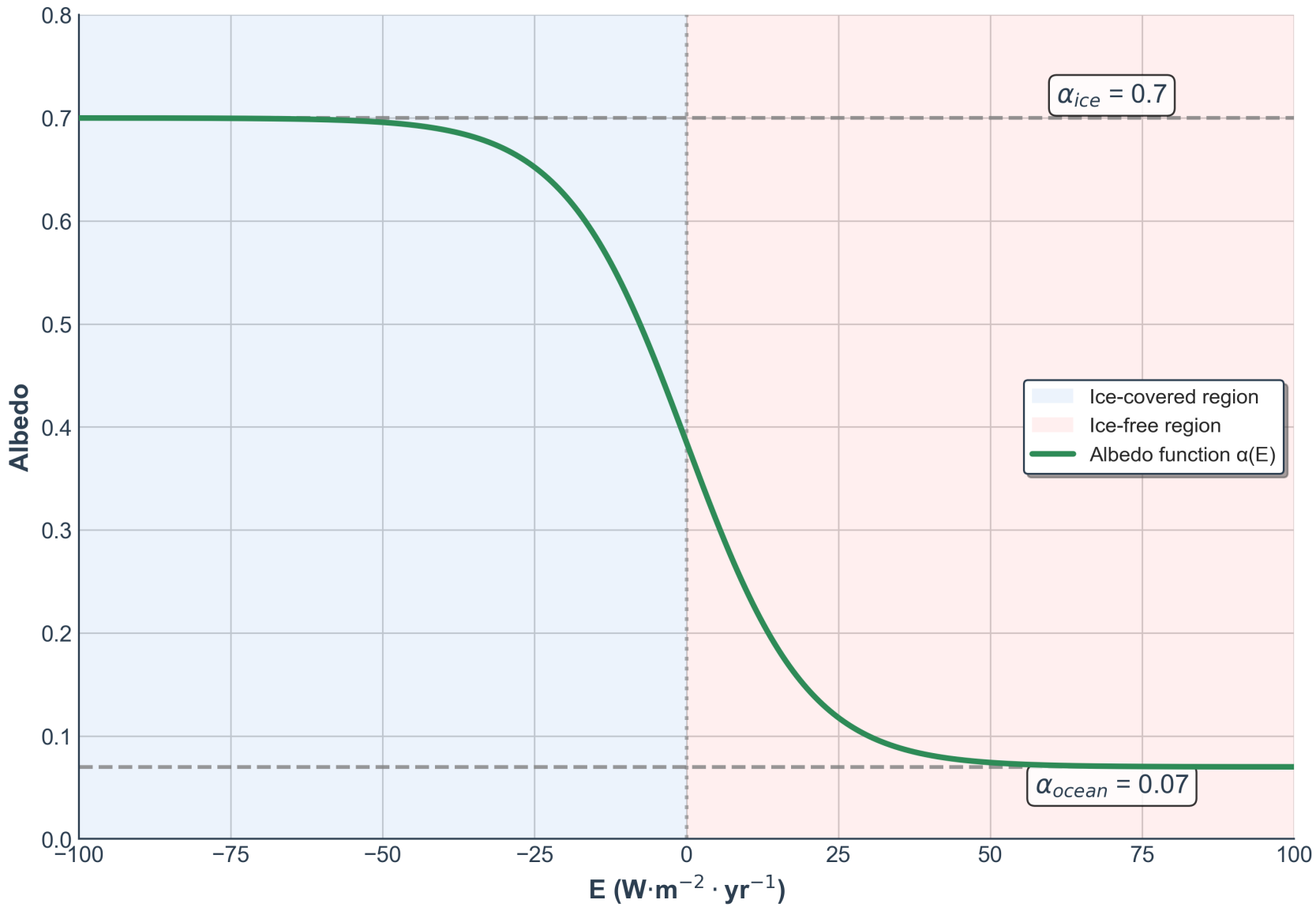


**Supplementary Figure 1:** Albedo as a function of energy $E$. The curve illustrates the transition from high albedo ($\alpha_{\text{ice}} = 0.7$) when the surface is ice-covered to low albedo ($\alpha_{\text{ocean}} = 0.07$) when the surface is ice-free.

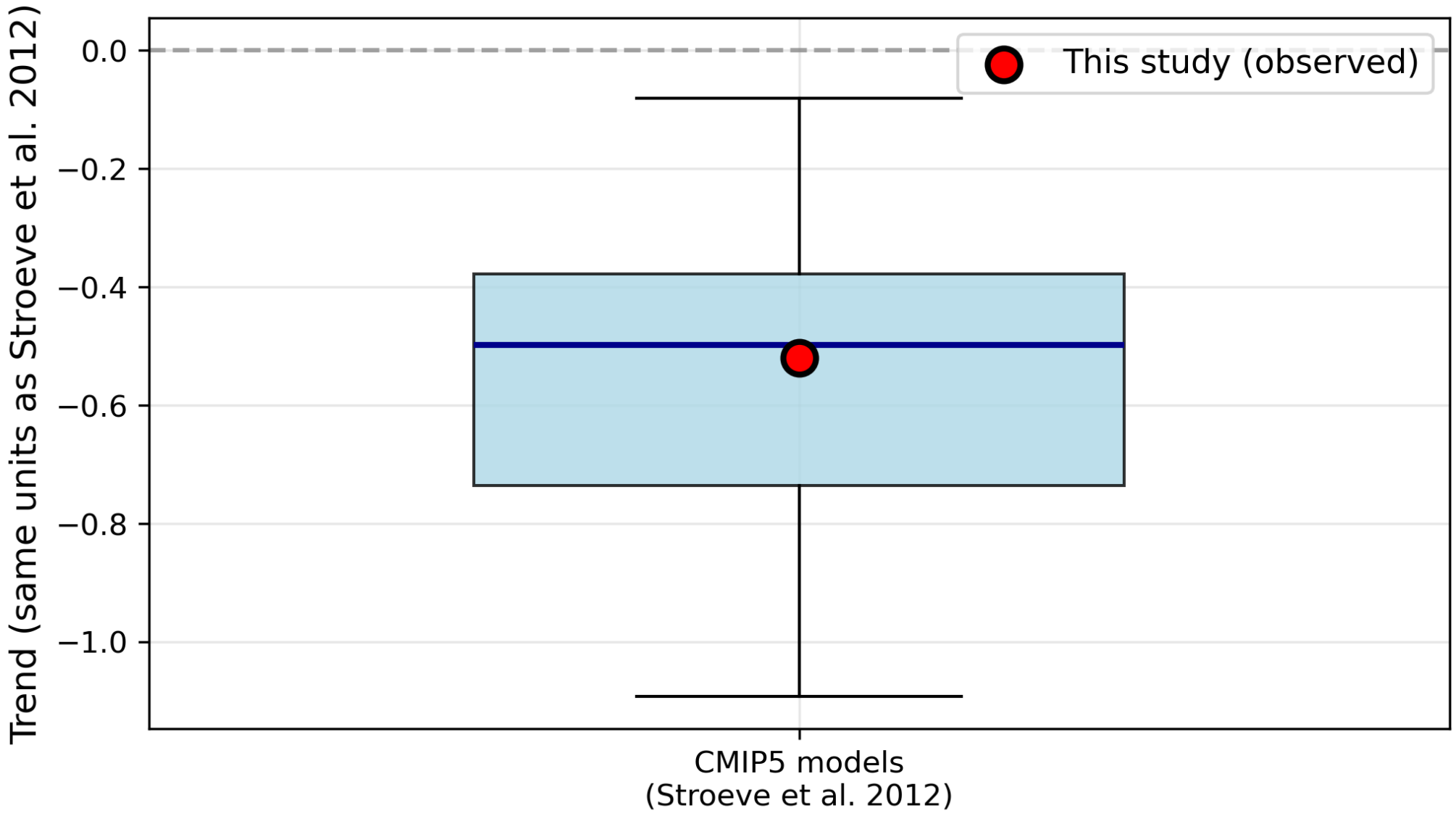


**Supplementary Figure 2:** Consistency check against published CMIP5 September sea ice extent trends. Distribution of 1979–2011 September SIE trends ($10^6$ km$^2$ per decade) across 20 CMIP5 models reported by Stroeve et al. (2012), shown as a boxplot, together with the observed trend from this study shown as a red point. The observed trend lies within the model distribution and close to the ensemble median; therefore, this comparison indicates broad consistency in sign and magnitude rather than a significant difference between observations and the model ensemble.

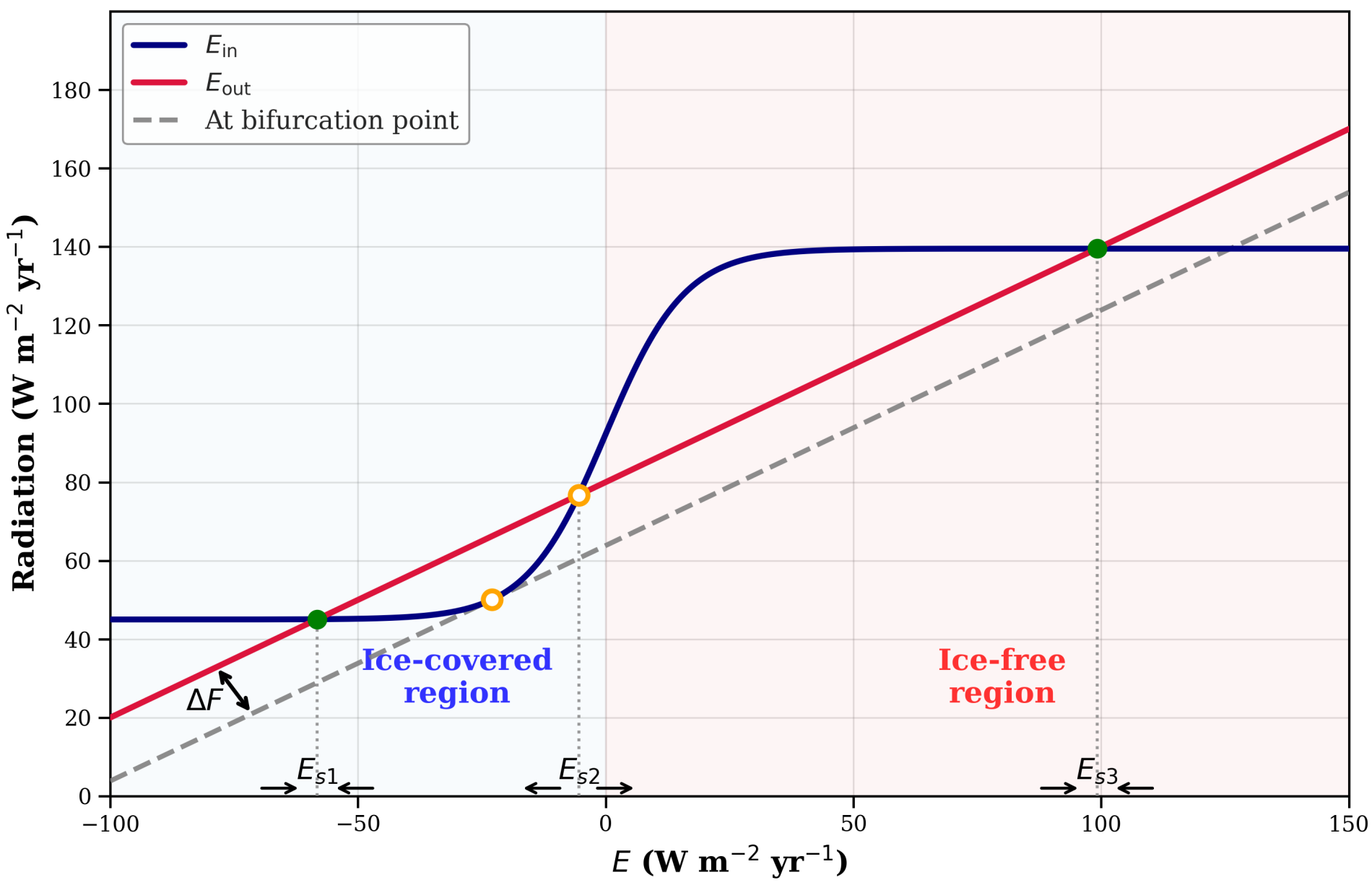


**Supplementary Figure 3:** The steady states ($E_{s1}$, $E_{s2}$, $E_{s3}$) occur where incoming radiation ($E_{\text{in}}$, blue curve) balances outgoing radiation ($E_{\text{out}}$, red line). The intersections at the extremes ($E_{s1}$, $E_{s3}$) are stable steady states, while the middle intersection ($E_{s2}$) is unstable. The dashed line illustrates the bifurcation point, where the ice-covered and unstable states merge at a tipping point.

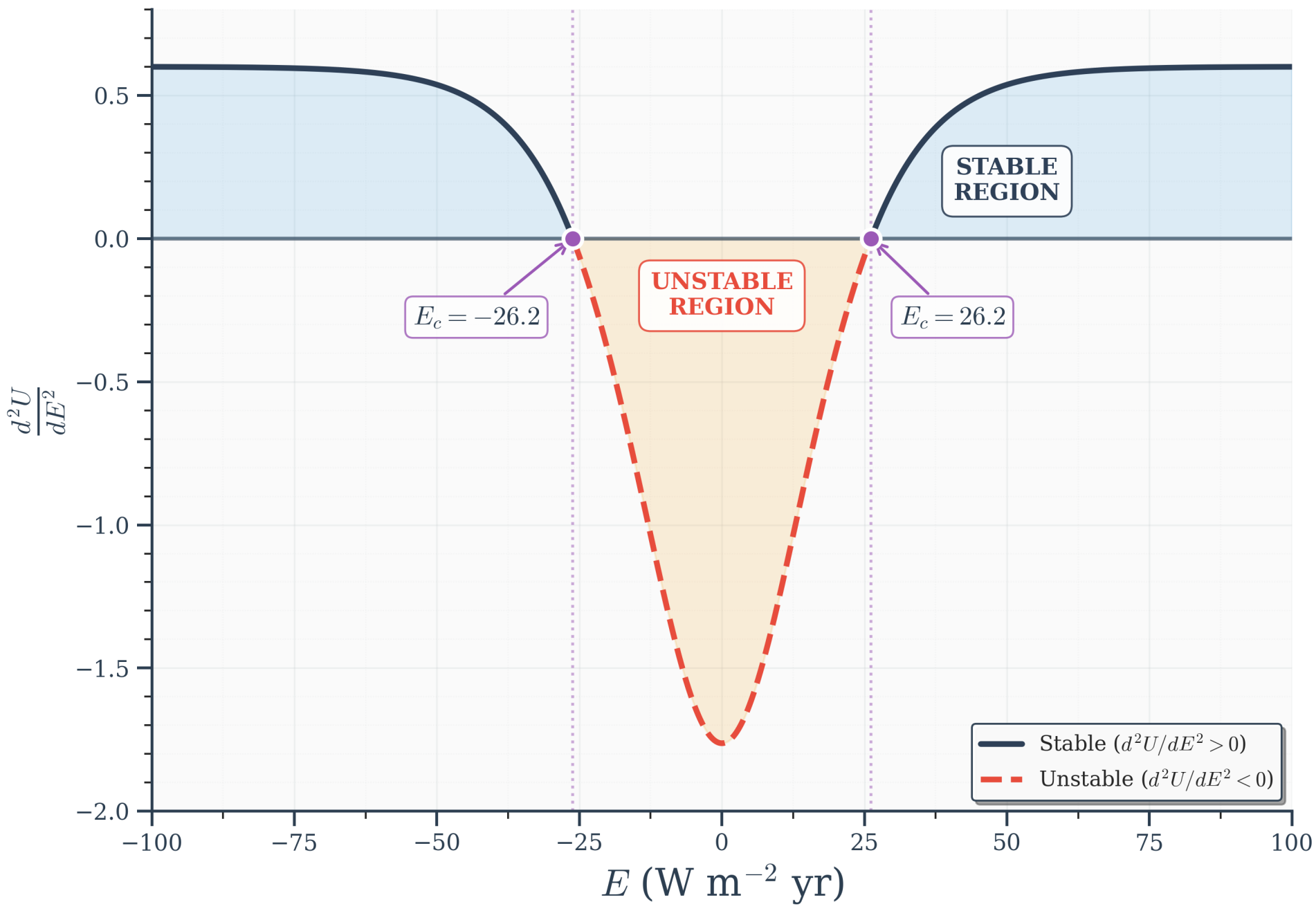


**Supplementary Figure 4:** Second derivative $U''(E) = d^2U/dE^2$ as a function of energy $E$ (example shown for $\Delta F \approx 0$). Positive values denote locally stable regions (fast recovery); negative values indicate instability near the saddle. Vertical dotted lines mark the zero-curvature crossings $E_c$.

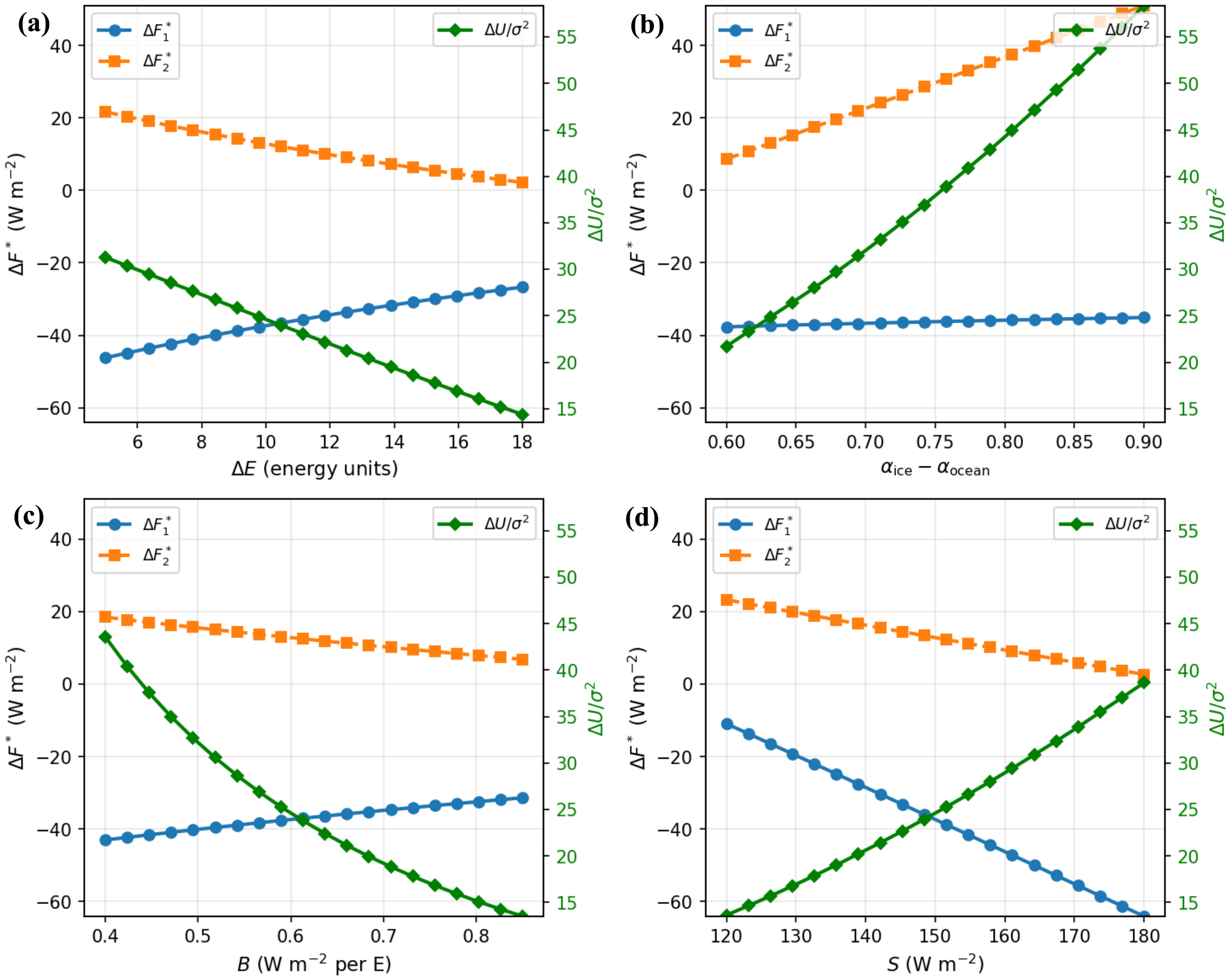


**Supplementary Figure 5:** Sensitivity of bifurcation points and barrier-to-noise ratio to reduced EBM parameters. Each panel shows one-at-a-time sensitivity of the lower and upper bifurcation thresholds, $\Delta F_1^*$ and $\Delta F_2^*$, and the model-defined barrier-to-noise ratio, $\Delta U/\sigma^2$, to changes in (a) transition width $\Delta E$, (b) albedo contrast $\alpha_{\rm ice} - \alpha_{\rm ocean}$, (c) longwave damping parameter $B$, and (d) effective shortwave forcing $S$. The tested ranges are physically plausible basin-mean perturbations around the baseline parameter set rather than formal observational uncertainty bounds. The figure shows that the qualitative reduced-model bifurcation structure is retained across the tested ranges, while the numerical threshold values and barrier metrics are parameter dependent.

**Supplementary Table 1:** Baseline parameter values and one-at-a-time sensitivity ranges used in the reduced energy-balance model. The tested ranges are intended to span physically plausible basin-mean values around the baseline state, rather than formal observational uncertainty bounds. See Methods for the rationale used to define these intervals.

| Parameter | Baseline | Range tested | Physical role | Rationale for tested range |
|---|---|---|---|---|
| $\Delta E$ | 10.0 | 5.0–18.0 | Smoothness of the ice–ocean transition in the sigmoid albedo function | Chosen to span relatively sharp to relatively smooth basin-mean ice–ocean transitions, while excluding unrealistically abrupt step-like behavior and overly diffuse transitions that strongly weaken the albedo feedback. |
| $\alpha_{\text{ice}} - \alpha_{\text{ocean}}$ | 0.63 | 0.60–0.90 | Effective ice–ocean albedo contrast controlling feedback strength | Chosen to span muted to strong basin-mean albedo contrast, from melt-affected ice conditions to bright ice/snow end-members commonly used in reduced Arctic EBM studies. |
| $B$ | 0.60 | 0.40–0.85 | Linearized longwave restoring strength | Chosen to bracket weak to strong effective radiative damping in reduced Arctic energy-balance models, while retaining an interpretable bifurcation structure. |
| $S$ | 150 | 120–180 | Effective absorbed shortwave forcing | Chosen to span weaker to stronger basin-mean shortwave input in the reduced framework, while preserving realistic Arctic radiative-balance scaling. |

**Supplementary Table 2:** Observationally estimated innovation scale $\sigma_E$ for October energy $E_{\text{obs}}$ in two analysis epochs. $\sigma_E$ is the standard deviation of the AR(1) innovation ($\sigma^2 = \text{Var}(E_{\text{obs}})(1-\phi^2)$) and is used in $\Delta U/\sigma^2$ and Kramers-type scaling. Values are consistent in order of magnitude with published basin-mean estimates of order 7 $\text{W m}^{-2}$ for the modern Arctic.

| Epoch (analysis window) | $\sigma_E$ ($\text{J m}^{-2}$) | $\sigma_E$ ($10^{12}\,\text{J m}^{-2}$) |
|---|---|---|
| 1982–2005 (October) | $1.688 \times 10^{11}$ | 0.1688 |
| 2006–2024 (October) | $1.806 \times 10^{11}$ | 0.1806 |

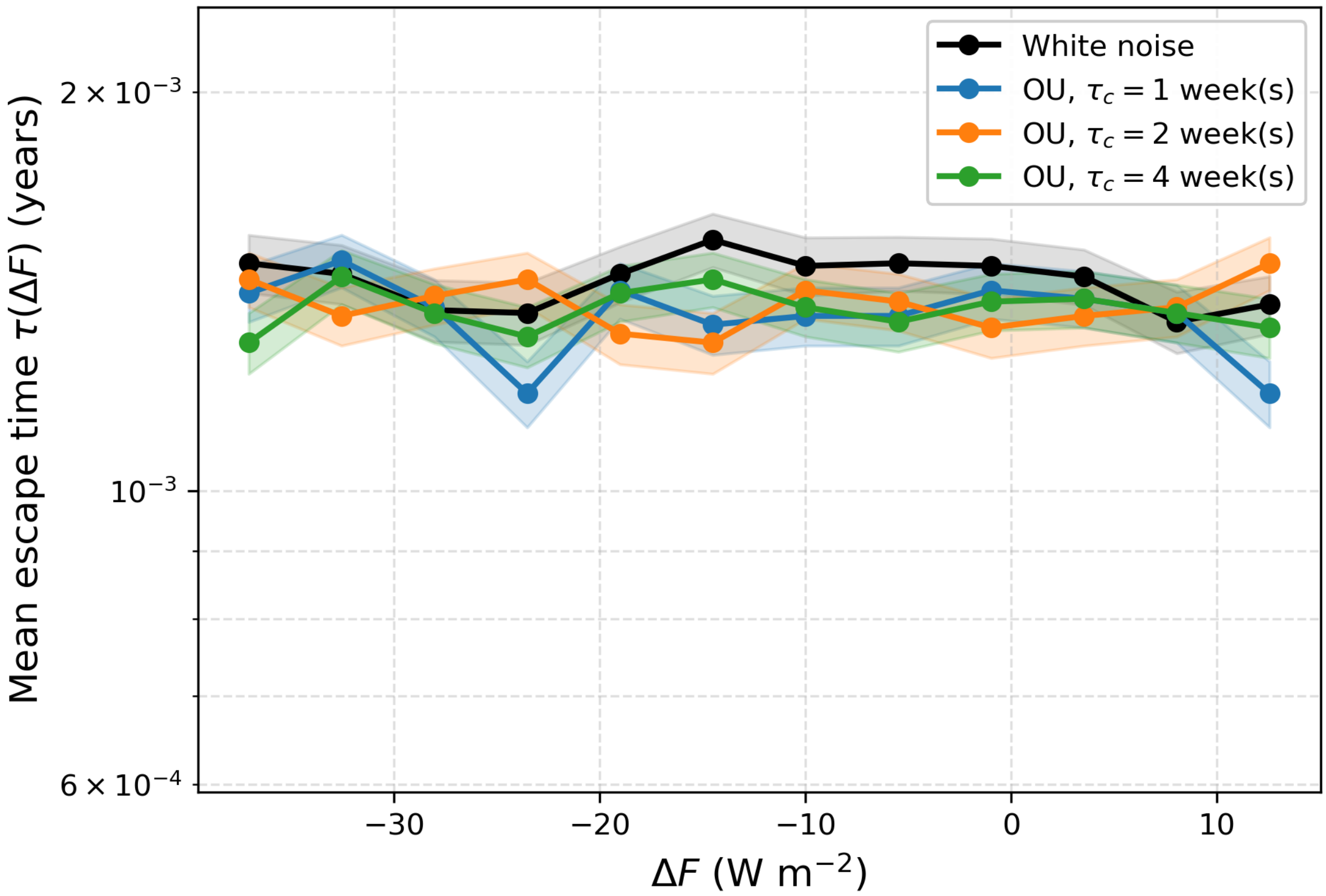


**Supplementary Figure 6:** Mean escape time $\tau(\Delta F)$ from the ice-covered minimum to the saddle in the reduced energy-balance model, for white noise (black) and Ornstein-Uhlenbeck forcing with decorrelation times $\tau_c = 1$, 2 and 4 weeks (blue, orange, green). The same marginal variance $\sigma^2$ is used in all cases. Shaded bands show $\pm 1$ standard error over realizations. The OU curves lie below the white-noise curve across the bistable range, illustrating that temporal persistence shortens the mean escape time for the same marginal variance.

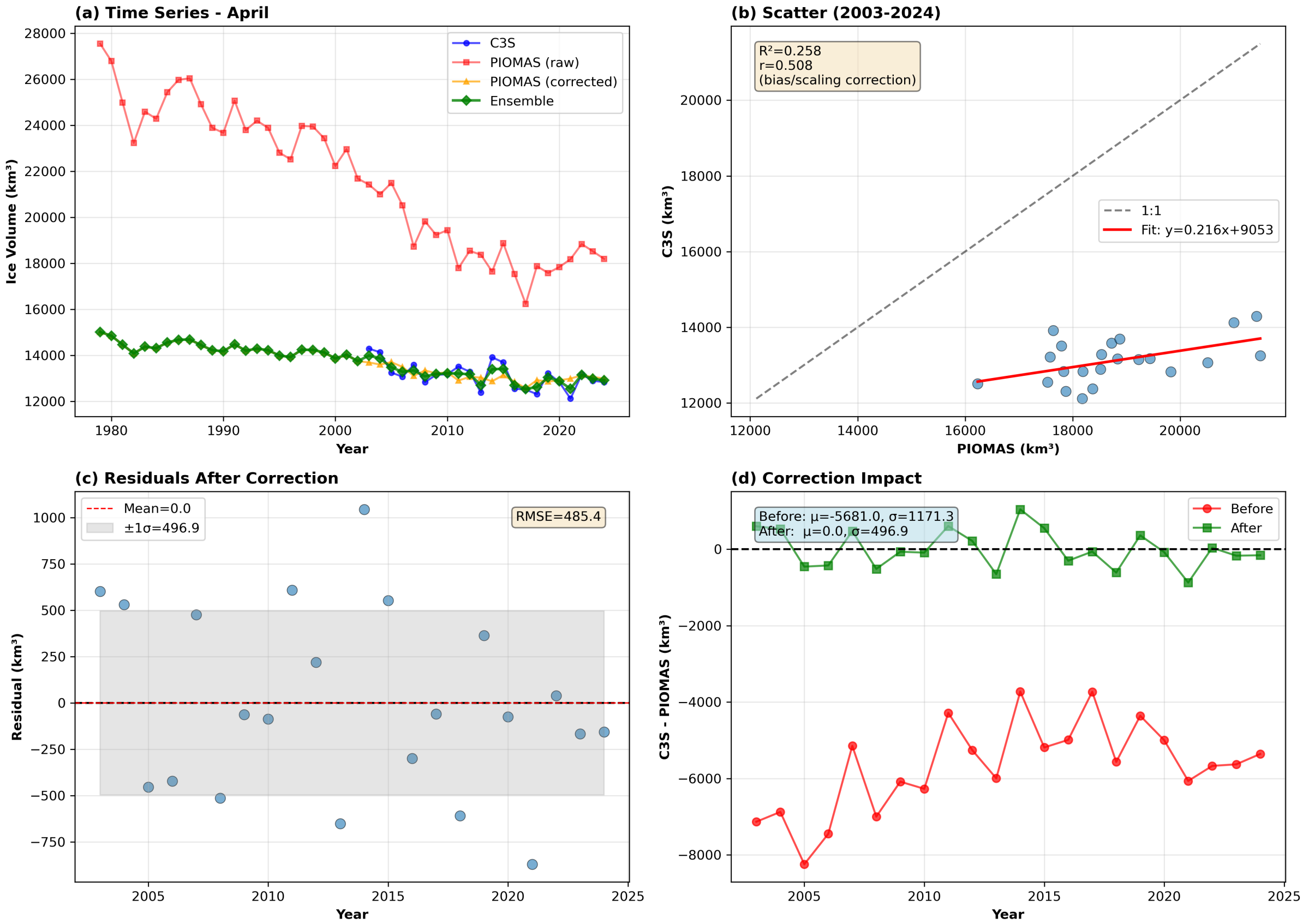


**Supplementary Figure 7:** (a) Time series of monthly Arctic sea ice volume for April from C3S (blue), raw PIOMAS (red), bias-corrected PIOMAS (orange), and the resulting ensemble mean (green). (b) Scatter plot of C3S versus PIOMAS over the overlap period (2003–2024) with fitted and 1:1 reference line (dashed). (c) Residuals of C3S - PIOMAS after correction, showing zero mean bias and RMSE. (d) Effect of bias correction on interannual differences: the mean offset ($\mu$ before correction) is effectively removed, and variability is reduced. The harmonization produces a stable composite volume record suitable for multi-decadal resilience diagnostics.

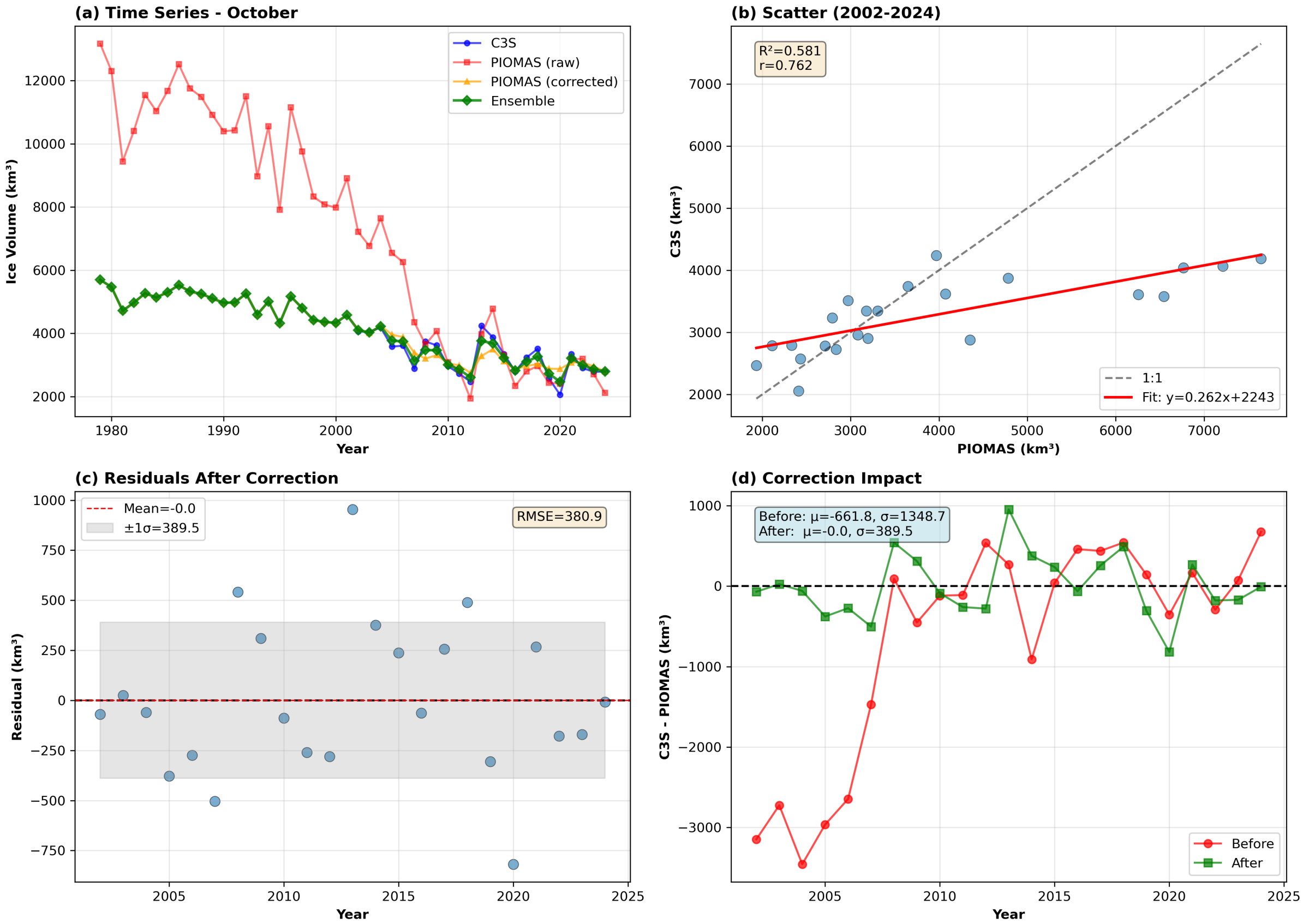


**Supplementary Figure 8:** Same as Supplementary Figure 7, but for October SIV harmonization.

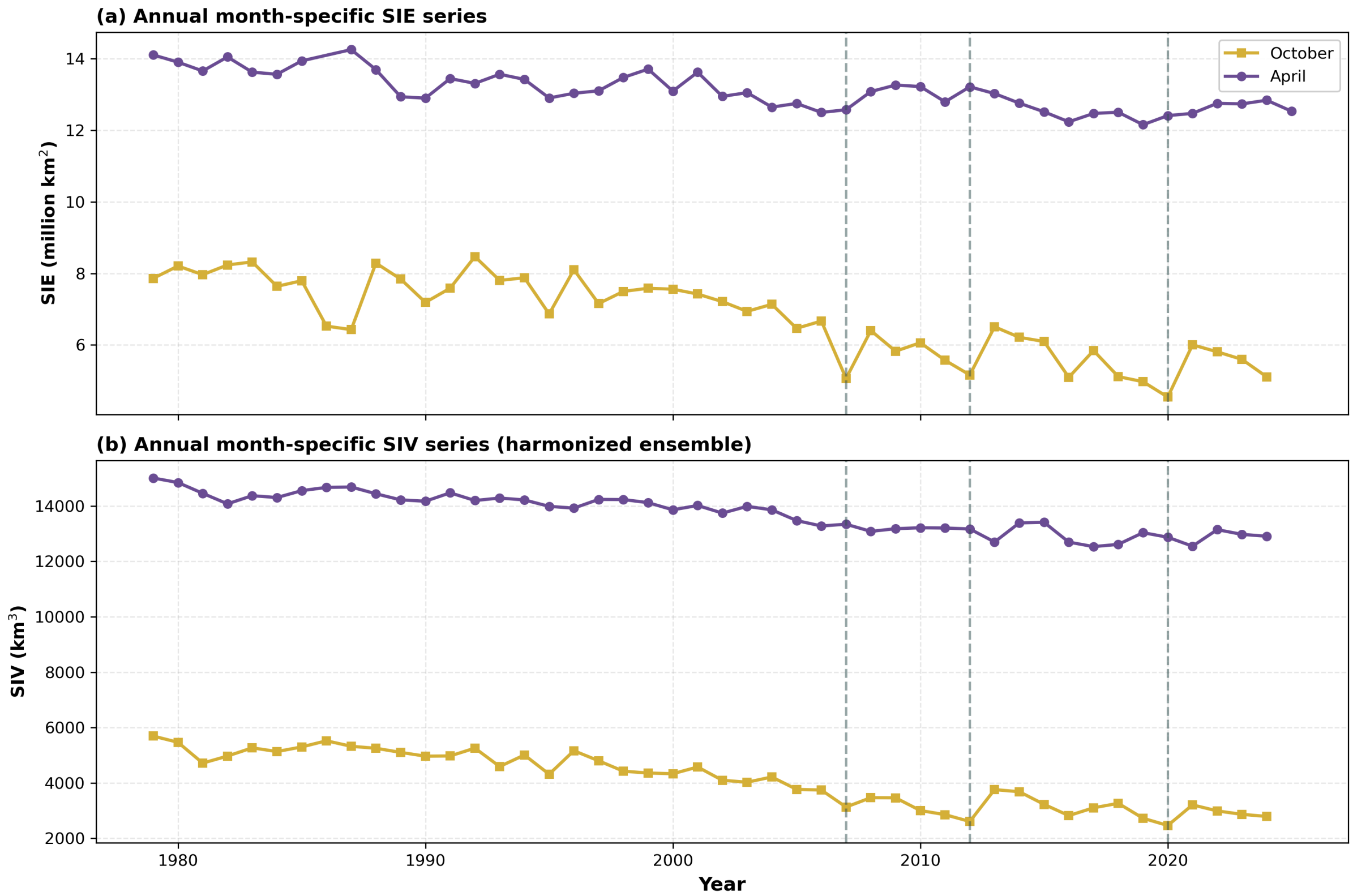


**Supplementary Figure 9:** Underlying annual April/October series used for AC1 diagnostics. (a) Annual month-specific Arctic sea ice extent (SIE). (b) Annual month-specific Arctic sea ice volume (SIV; harmonized ensemble of C3S and bias-corrected PIOMAS). Dashed vertical lines mark 2007, 2012, and 2020.

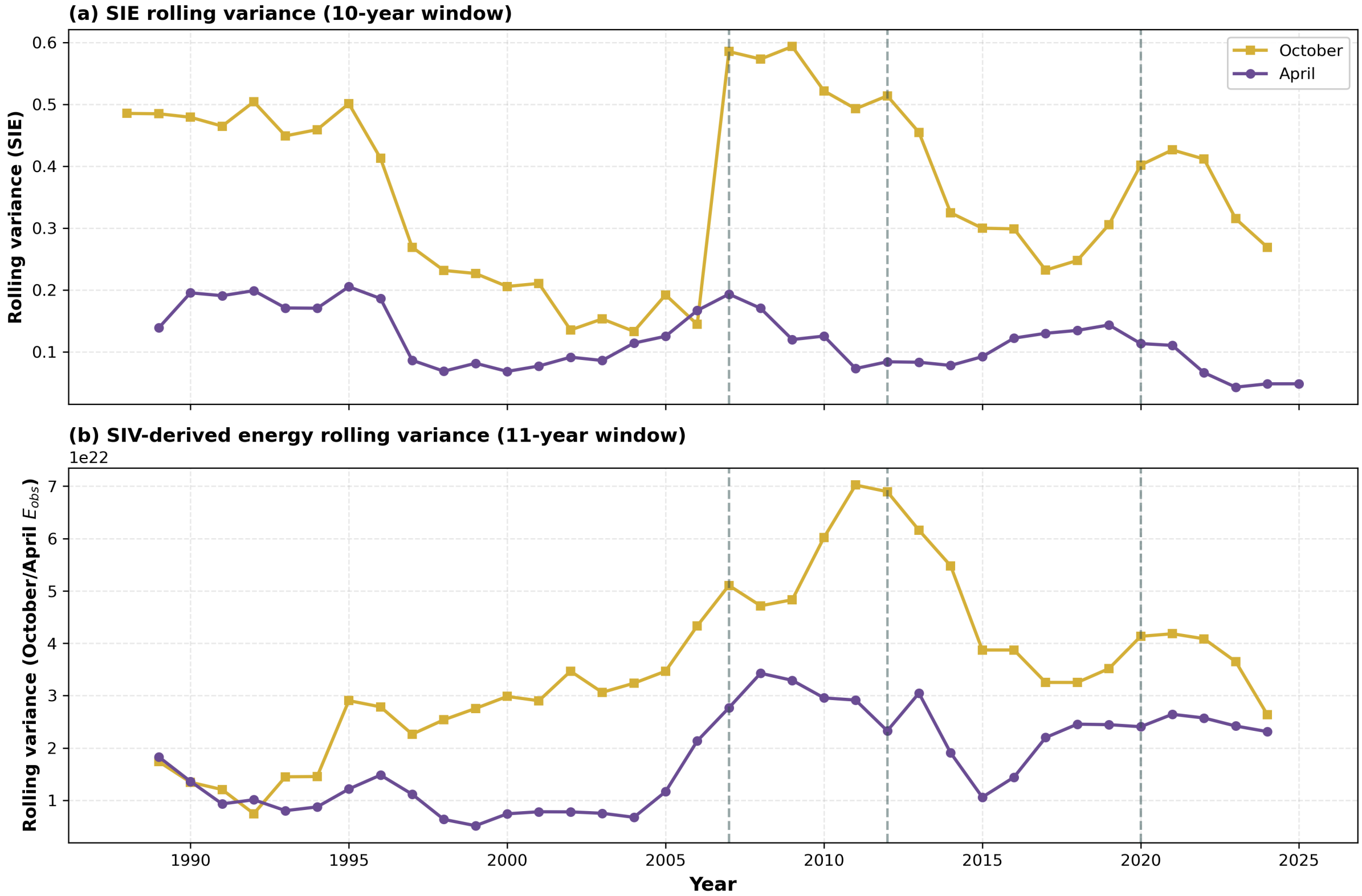


**Supplementary Figure 10:** Rolling variance diagnostics corresponding to the AC1 analysis windows. (a) SIE rolling variance (10-year window) for April and October. (b) Rolling variance of SIV-derived October/April energy state $E_{obs}$ (11-year window). Dashed vertical lines mark 2007, 2012, and 2020.
